# Genome-wide mapping of Sox6 binding sites in skeletal muscle reveals both direct and indirect regulation of muscle terminal differentiation by Sox6

**DOI:** 10.1186/1471-213X-11-59

**Published:** 2011-10-10

**Authors:** Chung-Il An, Yao Dong, Nobuko Hagiwara

**Affiliations:** 1 Division of Cardiovascular Medicine, Department of Internal Medicine, University of California, Davis, One Shields Avenue, Davis, California 95616, USA

## Abstract

**Background:**

Sox6 is a multi-faceted transcription factor involved in the terminal differentiation of many different cell types in vertebrates. It has been suggested that in mice as well as in zebrafish Sox6 plays a role in the terminal differentiation of skeletal muscle by suppressing transcription of slow fiber specific genes. In order to understand how Sox6 coordinately regulates the transcription of multiple fiber type specific genes during muscle development, we have performed ChIP-seq analyses to identify Sox6 target genes in mouse fetal myotubes and generated muscle-specific Sox6 knockout (KO) mice to determine the Sox6 null muscle phenotype in adult mice.

**Results:**

We have identified 1,066 Sox6 binding sites using mouse fetal myotubes. The Sox6 binding sites were found to be associated with slow fiber-specific, cardiac, and embryonic isoform genes that are expressed in the sarcomere as well as transcription factor genes known to play roles in muscle development. The concurrently performed RNA polymerase II (Pol II) ChIP-seq analysis revealed that 84% of the Sox6 peak-associated genes exhibited little to no binding of Pol II, suggesting that the majority of the Sox6 target genes are transcriptionally inactive. These results indicate that Sox6 directly regulates terminal differentiation of muscle by affecting the expression of sarcomere protein genes as well as indirectly through influencing the expression of transcription factors relevant to muscle development. Gene expression profiling of Sox6 KO skeletal and cardiac muscle revealed a significant increase in the expression of the genes associated with Sox6 binding. In the absence of the Sox6 gene, there was dramatic upregulation of slow fiber-specific, cardiac, and embryonic isoform gene expression in Sox6 KO skeletal muscle and fetal isoform gene expression in Sox6 KO cardiac muscle, thus confirming the role Sox6 plays as a transcriptional suppressor in muscle development.

**Conclusions:**

Our present data indicate that during development, Sox6 functions as a transcriptional suppressor of fiber type-specific and developmental isoform genes to promote functional specification of muscle which is critical for optimum muscle performance and health.

## Background

Skeletal muscle in vertebrates has evolved to be a major organ system with great adaptability in order to respond to constantly changing physical demands placed upon it. This adaptability is achieved by the ability of muscle fibers to change their contractile and metabolic properties. Adult skeletal muscle consists of two major fiber groups, slow-twitch and fast-twitch. In general, slow fibers are best fit for long-lasting aerobic activity whereas fast fibers are best fit for short bouts of anaerobic activity [1]. At the molecular level, a coordinated expression of multiple fiber type-specific genes, both structural and enzymatic, is required to give each fiber type its unique characteristics. Slow and fast muscle fibers are operationally defined by the expression of the isoforms of myosin heavy chain (MyHC) [2]. In adult rodent skeletal muscle, slow fibers are defined by the expression of MyHC-β, whereas fast fibers are defined by the expression of three MyHC isoforms, IIa, IIx/d, and IIb (contractive speed: IIa<IIx/d<IIb) [3]. In developing fetal rodent muscle, instead of MyHC-IIa, IIx/d, and IIb, there are two developmental MyHC isoforms (embryonic and perinatal) that are expressed, along with MyHC-β, at different stages of development [4,5]. After birth, expression of embryonic and perinatal MyHC isoforms as well as MyHC-β is significantly downregulated and the majority of the rodent muscle becomes fast MyHC-expressing fibers with exception of weight bearing core muscles such as soleus where slow MyHC-β is highly expressed [5-7]. In adult muscle, the main determinant of muscle fiber type is motoneuron input [3,8-11]. Several mediators and transcription factors have been identified for the nerve dependent fiber type regulation in adult skeletal muscle [3]. In contrast, our knowledge about factors that regulate fiber type differentiation during skeletal muscle development is still limited. We have previously reported that Sox6 mutant fetal and perinatal skeletal muscle exhibits a significant increase in slow fiber type-specific gene expression accompanied by a significant decrease in fast fiber type-specific gene expression [12,13]. Based on these observations, we have proposed that Sox6 functions as a transcriptional suppressor of slow fiber specific genes in developing skeletal muscle.

Sox6 is a member of the evolutionarily highly conserved Sox transcription factor family [14-17]. Between mice and humans, the overall amino acid sequence of the Sox6 protein is approximately 95% conserved, and the functional domains are 100% conserved [18]. The Sox proteins contain the Sry-related HMG box domain which mediates sequence-specific DNA binding [16,17]. In general, the specificity of Sox protein targets in each cell type is regulated by their cofactors [16,19], a property that is especially important for the Sox6 protein since it lacks a regulatory domain (activator or repressor). Therefore, when Sox6 is involved in transcriptional regulation, cofactors of Sox6 dictate whether the outcome is activation or repression [15,16]. For example, Sox6 activates cartilage specific gene transcription as part of the Sox trio proteins (Sox5, Sox6 and Sox9) [20-22]. In other cell types, Sox6 suppresses transcription of the fgf3 gene or the cyclinD1 gene by associating with repressors [23,24]. In the case of skeletal muscle, we have shown that Sox6 suppresses transcription of slow fiber specific genes during development, thus playing a critical role in initial muscle fiber type differentiation [12,13].

In the present study, to start to uncover how Sox6 regulates transcription of fiber type specific genes at the molecular level, we used a conditional Sox6 allele [25] to inactivate Sox6 in developing skeletal muscle. The muscle specific inactivation of Sox6 allowed us to overcome the perinatal lethality of Sox6 mutant mice [26,27] and obtain Sox6 knockout (KO) adult skeletal muscle for in-depth analysis. To identify Sox6 target genes and assess their transcriptional status, we conducted ChIP-seq analyses using Sox6 and RNA polymerase II (Pol II) antibodies. Combining these methods, we demonstrate that: (1) Inactivation of Sox6 results in an extreme upregulation in expression of slow fiber specific, cardiac and fetal isoform genes, suggesting that Sox6 is required for the functional maturation of skeletal muscle, and (2) Sox6 binds to the DNA sequences in the vicinity of these genes, and thus is directly involved in the transcriptional suppression of its target genes. These results indicate that Sox6 plays a critical role in functional specification of muscle during development.

## Results

### The expression level of MyHC-β is dramatically increased in Sox6 KO muscle during development

We have previously shown that in the Sox6 null fetal skeletal muscle, nascent fast muscles maintain slow MyHC-β expression [13]. In addition to MyHC-β, other slow fiber specific genes (e.g. *Tnnc1, Tnni1, Tnnt1*, and *Myl2*) are also upregulated in the Sox6 null muscle, along with significant downregulation of multiple fast fiber specific genes [12,13]. Based on these results, we proposed that Sox6 functions as a suppressor of slow fiber specific genes, thus the loss of Sox6 leads to an increase in slow muscle fibers. Since Sox6 null mutations cause early postnatal lethality [26,27], we were unable to determine whether this Sox6 null fetal phenotype is maintained through postnatal development. To overcome the lethal phenotype, we utilized mice carrying a Sox6 conditional allele [25] to inactivate Sox6 specifically in skeletal muscle. To start assessing the phenotype of adult Sox6 KO muscle, we first used the Myf5-Cre mouse [28]. In this Cre-transgenic mouse, the Cre recombinase under the control of the Myf5 promoter is expressed very early in the skeletal muscle lineage (starting at approximately E8 in somites); therefore, the inactivation of Sox6 occurs significantly earlier than the beginning of fiber type specification [4,5]. To conduct a comprehensive analysis of the Sox6 KO muscle phenotype, we examined four different muscles in the hindlimb, the tibialis anterior (TA, fast), extensor digitorum longus (EDL, fast), gastrocnemius (fast), and soleus (slow) [6]. The mRNA expression of the following four genes: slow MyHC-β (*Myh7*), fast MyHC-IIb (*Myh4*), peroxisome proliferative activated receptor γ coactivator 1α (*Ppargc1a*), and succinate dehydrogenase complex subunit A (*Sdha*) were determined by reverse transcription-quantitative PCR (RT-qPCR) and compared between Sox6 KO (Sox6^f/f^; Myf5-Cre) and control (Sox6^f/f^) mice. As summarized in Table 1, Sox6 inactivation caused a significant increase in the mRNA expression of *Myh7 *and a concurrent decrease in *Myh4 *in the TA, EDL, and gastrocnemius muscles. The Sox6 KO soleus muscle showed the least change in expression of these two MyHC isoforms (Table 1). This result likely reflects the observation that Sox6 expression in soleus is significantly lower than the other three fast muscles (Additional file 1, Figure S1A), therefore, Sox6 inactivation may have had a less impact in soleus compared to the other muscles. Regarding the Sox6 inactivation levels in adult muscle, we noticed that a higher level of Sox6 inactivation, determined by Sox6 mRNA level, did not necessarily correlate with an increase in Myh7 mRNA level. There are a few possible hypotheses to explain this observation. First, Sox6 is not a muscle specific gene and is also expressed in fibroblasts, which can obscure an accurate quantification of Sox6 mRNA specific to muscle cells. Second, the Sox6 mutation is recessive in nature. Therefore, although two independent Sox6 KO muscle samples show 50% reduction in Sox6 mRNA level, one sample may have more homozygous Sox6 null cells and the other may have more heterozygous cells, leading to a significant difference in Myh7 expression. Third, skeletal muscle is multinucleated, which adds another layer of complexity as to how Sox6 inactivation in each nucleus influences Myh7 expression in a myotube as a whole.

**Table 1:** Fold change in mRNA levels in the Sox6 KO skeletal muscles compared to control

	TA		EDL		Gas		Sol	
	
Mouse age (month)	2	3	2	3	2	3	2	3
Sox6	0.51	0.10	0.94	0.01	0.12	0.20	0.36	0.48
Myh7 (MyHC-β)	779.78	147.72	22.36	4186.18	5.75	11.24	1.80	2.71
Myh4 (MyHC-IIb)	0.03	3 × 10^-3^	0.20	3 × 10^-3^	0.12	2 × 10^-3^	0.88	0.30
Ppargc1a (PGC1-α)	3.27	0.35	2.06	0.13	0.22	0.61	0.85	1.92
Sdha	1.02	0.38	1.93	0.68	0.97	0.51	1.00	1.27

Sox6 was inactivated using Myf5-Cre mice. A two month-old and a three month-old mice were examined. Control expression level = 1.00.

**Figure 1:**
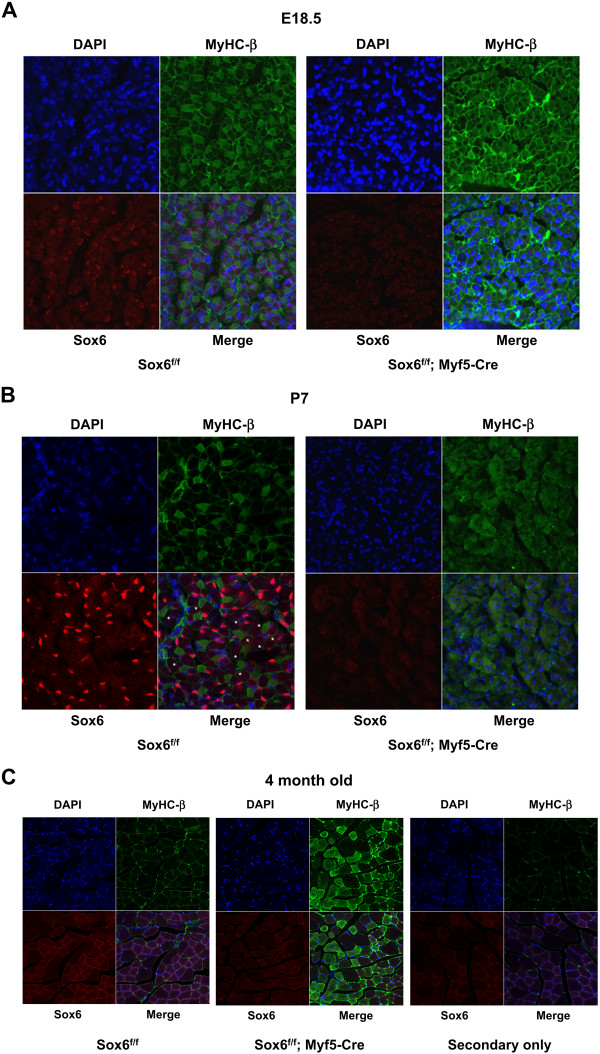
**The number of fibers expressing MyHC-β is dramatically increased in Sox6 KO skeletal muscle**. A. Cross-sections of E18.5 lower hindlimb muscle (TA-EDL region) from control (Sox6^f/f^) and Sox6KO (Sox6^f/f^; Myf5-Cre) were stained with DAPI (blue) or specific antibodies for MyHC-β (green) or Sox6 (red). x400 magnification. B. P7 (7 day old) hindlimb muscle processed for DAPI, Sox6 and MyHC-β immune-staining.* indicates myotubes that are negative for both MyHC-β and Sox6 staining in control muscle (see text for discussion). x400 magnification. C. Four month old adult TA-EDL muscle. A control muscle section stained with DAPI or secondary antibodies (a mixture of both anti-mouse and anti-rabbit) only was also shown. ×200 magnification.

To sort out these issues, we performed immunohistochemistry to examine the Sox6 and Myh7 (MyHC-β) protein expression at the cellular level in fetal, early postnatal and adult muscle. We focused our observation on the TA-EDL region, composed of fast-twitch myofibers in the adult mouse. As shown in Figure 1A, in E18.5 control (Sox6^f/f^), nuclear Sox6 staining was well correlated with the absence of cytoplasmic MyHC-β staining. Also at P7, the presence of Sox6 nuclear staining corresponded to MyHC-β negative myotubes (Figure 1B). In Sox6 KO muscle (Sox6^f/f^; Myf5-Cre), at both stages, nearly 100% of myofibers displayed MyHC-β expression (Figures 1A and 1B). These data show that Sox6 expression does not coincide with slow-twitch fiber gene expression, supporting our idea that Sox6 functions as a suppressor of the slow-twitch fiber gene program. Therefore, at the protein level, the loss of Sox6 expression clearly leads to upregulation of MyHC-β during the early stages of muscle development. During the normal mouse fast muscle development, the number of MyHC-β positive slow-twitch fibers significantly decreases as postnatal skeletal muscles functionally mature [3]. We observed this trend in the developing control mouse muscles (Figures 1A and 1B), resulting in adult TA-EDL muscle with extremely rare MyHC-β positive myofibers (Figure 1C). In contrast to the control, at E18.5 and P7, nearly all Sox6 KO myofibers were MyHC-β positive (Figures 1A and 1B), indicating that at these early stages, muscle-specific Sox6 inactivation led to extensive upregulation of MyHC-β expression in the entire Sox6 KO muscle. In the adult Sox6 KO muscle, on the other hand, approximately 50% of myofibers were MyHC-β positive, a significant increase compared to the control (Figure 1C, Sox6^f/f^); however a significant decrease compared to the P7 Sox6 KO muscle (Figure 1B, Sox6^f/f^; Myf5-Cre). When Sox6 staining signals in the control and Sox6 KO adult muscles were compared, overall Sox6 staining signals were lower in Sox6 KO, however, it was hard to make a clear correlation with MyHC-β staining, since Sox6 staining in adult muscle was quite diffused (Figure 1C, Sox6^f/f^). In light of this, we noticed that in control P7 muscle, some MyHC-β-negative myofibers did not show nuclear Sox6 staining, but rather dispersed cytoplasmic Sox6 staining (Figure 1B, marked with * in Sox6^f/f^). This observation may suggest an unknown additional mechanism to relocate the Sox6 protein from the nucleus and/or degrade it in differentiated, more mature myotubes. A recent report on Six1/Six4 double KO muscle suggests that these two proteins positively regulate fast-twitch fiber differentiation and may also influence Sox6 nuclear localization during fetal muscle development (E18.5) [29]. In adult muscle, therefore, not only Sox6 expression, but other mechanisms such as the Six1/Six4 regulated Sox6 shuttling may be in place to finalize fiber type gene expression in response to the environmental cues.

In addition to the muscle structural protein genes, we also examined mRNA expression of the genes playing a role in muscle metabolism, *Ppargc1a *(PGC-1α) and *Sdha *in adult muscle. Ppargca1 is a co-regulator of mitochondrial biogenesis and oxidative phosphorylation [30,31] and Sdha is a component of TCA cycle and complex II of the mitochondrial respiratory chain, whose expression is activated by Ppargc1a [30]. We speculated that *Ppargca1 *and *Sdha *mRNA expression would also be upregulated in Sox6 KO muscle, because of a correlation between oxidative metabolism and slow fiber content reported in adult skeletal muscle [1,32,33]. In spite of this expectation, neither *Ppargc1a *nor *Sdha *mRNA showed a noticeable increase in the Sox6 KO muscles (Table 1). This lack of correlation of the two gene programs was also observed in Sox6 KO muscles generated using MCK-Cre transgenic mice (Table 2, discussed later in the text). In a recent report on the adult Sox6 KO muscle phenotype, Quiat et al. also reported that expression of *Ppargc1a *was not changed [34]. Therefore, these results suggest that Sox6 plays a role in transcriptional regulation of the structural protein genes which define muscle fiber types, but not of the genes which define the metabolic state of skeletal muscle. In order to uncover the mechanisms of muscle differentiation that are regulated by Sox6 at the molecular level, we next performed Sox6 ChIP-seq analysis.

**Table 2:** Fold change in mRNA levels in the Sox6 KO skeletal muscles compared to control

	TA			EDL			Gas			Sol		
	
Mouse ID#	1	2	3	1	2	3	1	2	3	1	2	3
**Sox6**	0.75	0.42	0.09	0.43	0.38	0.07	0.38	0.53	0.10	0.20	0.31	0.12
**Myh1 (IIx/d)**	2.08	1.68	0.20	1.31	5.77	5.49	6.79	14.80	2.11	0.01	0.03	1 × 10^-3^
**Myh2 (IIa)**	5.00	1.96	0.80	3.08	2.11	4.72	6.63	5.77	1.85	0.02	0.02	u.d.
Myh4 (IIb)	0.17	0.01	0.01	0.11	0.04	0.02	0.25	1 × 10^-3^	0.02	0.16	0.16	0.01
***Myh6 *(**α**)**	78.79	252.99	1.03	24.19	25.37	6.65	3.54	4.13	2.42	4.03	1.61	0.65
***Myh7 *(**β**)**	9042.52	2177.81	94.12	1318.30	728.63	1611.29	21.86	3.59	9.68	3.60	1.00	1.20
***Myh7b***	31.37	n.d.	0.49	3.98	n.d.	1.39	14.30	n.d.	3.06	1.51	n.d.	0.34
***Myl2***	1496.57	n.d.	7.07	57.22	n.d.	55.59	7.37	n.d.	2.70	1.91	n.d.	1.31
***Tnnc1***	6830.19	4622.47	474.50	665.43	687.83	128.70	91.24	167.58	14.52	0.81	2.98	1.53
***Tnni1***	2619.20	1867.14	610.53	433.38	552.33	861.06	116.58	69.76	24.71	2.64	2.58	2.59
Tnni2	0.34	0.50	0.20	0.36	0.89	0.15	0.46	0.76	0.35	0.01	0.14	2 × 10^-3^
***Tnnt1***	4348.43	1594.44	157.77	1011.39	510.51	153.18	131.98	59.18	5.07	2.93	1.76	1.56
***Tnnt2***	2.45	1.88	2.08	2.31	0.59	1.55	1.23	2.65	1.37	2.59	1.12	1.85
Tnnt3	0.45	0.40	0.43	0.28	0.89	0.58	0.34	0.38	0.19	4 × 10^-3^	0.06	2 × 10^-4^
***Chrng *(fetal)**	0.89	4.63	4.94	0.73	1.80	1.82	1.23	1.80	3.29	2.78	1.17	8.26
Chrne (adult)	0.44	0.30	0.28	0.14	0.18	0.76	0.16	0.15	0.14	0.24	0.34	1.44
***Prox1***	29.26	10.30	9.66	22.84	6.68	17.04	16.21	8.41	3.34	3.79	2.26	1.27
**Tead1**	1.83	n.d.	0.99	0.63	n.d.	0.98	1.25	n.d.	0.78	1.02	n.d.	0.94
**Tead4**	3.03	n.d.	1.26	1.34	n.d.	1.08	1.23	n.d.	1.08	0.96	n.d.	1.11
**Tcf4**	1.94	n.d.	1.70	0.88	n.d.	1.54	1.68	n.d.	1.15	0.88	n.d.	1.43
**Hdac9**	4.44	n.d.	2.68	1.51	n.d.	1.79	7.56	n.d.	1.42	1.34	n.d.	0.75
Myod1	0.73	1.01	0.70	0.73	1.02	2.11	1.47	1.44	1.17	1.51	1.77	0.72
*Myog*	3.64	1.52	2.24	1.98	1.84	3.00	2.61	1.12	0.95	4.48	1.89	1.35
**Mb**	1.84	1.47	1.48	0.66	2.30	1.75	1.88	3.50	1.54	0.77	1.62	1.09
Sdha	1.18	0.74	0.39	0.70	0.74	0.50	0.79	0.60	0.32	0.68	0.59	0.55
**Ppargc1a**	1.28	1.39	0.53	0.82	0.78	0.35	0.63	1.46	0.42	0.53	0.71	0.19

*Sox6 *was inactivated using MCK-Cre mice. Mouse 1 and mouse 2 are two month-old, and mouse 3 is three month-old. Gene names in bold: associated with Sox6 binding. Gene names in italics: slow fiber specific sarcomere proteins, or transcription factors reported that are preferentially expressed in slow fibers. n.d.: not determined. u.d.: undetected in Sox6 KO mouse. Control expression level = 1.00.

### Genome-wide Sox6 binding in skeletal myotubes

**Figure 2:**
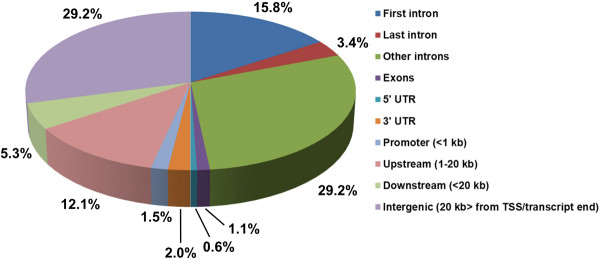
**Genome-wide mapping of Sox6 binding sites by ChIP-seq**. Locations of Sox6 binding sites relative to the nearest RefSeq genes and the percentages of binding sites at the respective locations are shown.

To identify genome-wide binding of Sox6 in mouse skeletal muscle, we performed ChIP-seq analysis. As the chromatin source, we chose wild type fetal (E.18.5) myotubes differentiated for 48 hours in vitro, because at this time point, a significant differential expression of slow fiber specific genes was observed between Sox6 null and wild type myotubes [13], suggesting an ideal time point to capture Sox6 acting as a transcriptional suppressor of those genes. Also, since Sox6 is highly expressed in fibroblasts (unpublished data), using a pure muscle cell population was necessary to identify muscle-specific Sox6 binding. We conducted two independent ChIP-seq experiments and obtained 3 and 1.5 million reads unambiguously mapped to the mouse genome for each experiment (out of ~20 million total reads). As a result, we identified 1,066 Sox6 peaks common to the two ChIP-seq data sets. These peaks were assigned to a total of 867 mouse RefSeq genes. The vast majority of the Sox6 binding sites were located in intronic regions (48.4%), followed by intergenic regions (more than 20 kb away from transcription start site (TSS) or transcript end) (29.2%) and 5'-upstream region (within 20 kb of TSS including promoter) (13.6%) (Figure 2).

**Figure 3:**
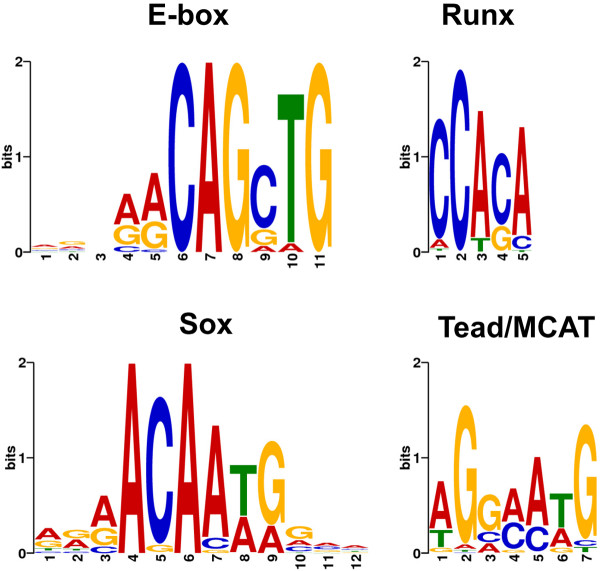
**Transcription factor consensus motifs found in Sox6 binding peaks**.

To determine whether any known transcription factor consensus sequences are over-represented within the Sox6 peak regions, a motif search was performed. Motif analysis using MEME (Multiple EM for Motif Elicitation) [35] identified four known transcription factor consensus motifs in the Sox6 peaks (Figure 3). When the occurrence of a single motif was set to 0 or 1 per peak, 723 Sox motifs (*P *< 10^-4^) and 636 E-box motifs (*P *< 10^-4^) were identified. The fact that the Sox consensus motifs were found in the overwhelming majority of the Sox6 peaks (723 out of 1,066) suggests that the Sox6 binding sites identified here are bona fide Sox6 targets. The E-box motifs (CAG[C/G]TG) identified using the *in silico *method here were identical to the E-box motifs which were enriched in MyoD binding sites detected using C2C12 myotubes [36]. Comparing our data with the MyoD ChIP-seq data obtained from adult mouse primary myotubes [36] revealed that 96% of the Sox6 peaks were localized within 50 bp of the MyoD peaks (data not shown).

In addition to Sox motif and E-box, Runx and Tead/MCAT motifs were also found in the Sox6 peaks. When the occurrence of a single motif was set to 1, we identified 559 Runx motifs (*P *< 10^-4^) and 203 Tead/MCAT motifs (*P *< 10^-4^). A recent report has shown that Runx1 has a role in skeletal muscle terminal differentiation [37]; therefore, Runx transcription factors might be involved in muscle specific gene expression together with Sox6. Tead/MCAT elements are known to play an important role in transcriptional regulation of many skeletal and cardiac muscle-specific genes [38]. A significant presence of Tead/MCAT motifs in the Sox6 peaks, therefore, implies possible interactions between Sox6 and the Tead transcription factors during muscle differentiation.

### Transcriptional status of the genes associated with Sox6 binding sites

**Figure 4:**
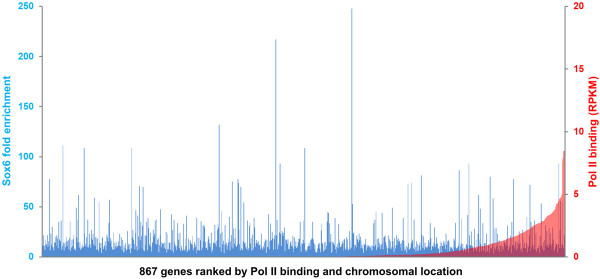
**Comparison of Sox6 binding and Pol II binding to the Sox6 target genes**. Left Y axis shows fold enrichment of Sox6 obtained by the peak calling program SISSRs using the 3 million read data set (see the text for detail), and right Y axis shows Pol II binding levels to the Sox6 peak-associated genes measured in RPKM. X axis shows all Sox6-associated genes (867 RefSeq genes in total) sorted according to Pol II binding and chromosomal location. When multiple Sox6 peaks were associated with one gene, only the peak with the highest fold enrichment was used. Note that Pol II binding to β-actin, an abundantly expressed housekeeping gene, was 8.60 RPKM. A similar result was obtained using the 1.5 million read-data set (data not shown).

In order to determine the transcriptional status of Sox6 peak-associated genes in differentiating fetal myotubes, we performed ChIP-seq analysis using an antibody recognizing a phosphorylated form of Pol II, which is considered to be a transcriptionally active form and associated with highly transcribed genes [39]. To quantify Pol II binding levels of RefSeq genes associated with Sox6 binding sites, Pol II binding events in the corresponding gene regions were measured in RPKM (reads per kilobase of gene region per million reads), a unit used to quantify transcriptional levels in RNA-seq analysis [40]. RPKM was calculated from read (tag) numbers in peak regions, length of RefSeq gene regions, and total number of uniquely mapped reads (details in Methods). By this method, the Pol II binding level of the β-actin gene, an abundantly expressed housekeeping gene, was calculated as 8.60 RPKM. Figure 4 summarizes the fold enrichment of the Sox6 peaks and the corresponding Pol II binding of the 867 RefSeq genes associated with Sox6 peaks. We found that the majority of the Sox6 binding site(s)-associated genes were inferred to be transcriptionally inactive (zero to a very low level of Pol II binding). As shown in Figure 4 andAdditional file 2, Table S1, of the 867 genes associated with Sox6 binding sites, 442 genes (51%) showed no Pol II binding (0 RPKM) and 289 genes (33%) showed less than one tenth of the Pol II binding to the β-actin gene (<0.86 RPKM), thus 84% of the genes associated with Sox6 binding sites are considered to be transcriptionally inactive or transcribed at a very low level in myotubes. These data strongly suggest that the binding of Sox6 to its targets mostly results in transcriptional suppression. The rest of the Sox6 peak associated genes were transcribed mostly at a range of low to moderate levels (less than half of the Pol II binding to the β-actin gene). There were, however, a small number of Sox6 peak-associated genes that exhibited a high level of Pol II binding. For example, *Myl4 *(embryonic MyLC isoform), *Tnnc1*, and *Myh3 *(embryonic MyHC isoform) showed a relatively high level of Pol II binding (>4.30 RPKM). In the case of *Tnnc1*, one of the two Sox6 peaks was identified in the first intron (Additional file 3, Figure S2D), where a muscle enhancer element was reported [41]. Therefore, an unidentified enhancer element may exist in the vicinity of the Sox6 binding sites in *Myl4 *and *Myh3*.

### Functional characterization of the genes associated with Sox6 binding sites

Gene Ontology (GO) analysis revealed that the Sox6 peak-associated genes showed the highest enrichment for the GO categories relevant to muscle cytoskeleton and myofibril establishment (Table 3). Many of these genes encode muscle sarcomeric proteins which define fiber types, cardiac isoforms, and developmental isoforms in muscle. For instance, *Myh1 *(fast MyHC-IIx/d), *Myh2 *(fast MyHC-IIa), *Myh6 *(cardiac isoform, MyHC-α), *Myh7 *(slow MyHC-β), *Myh7b *(myosin, heavy chain 7B, cardiac muscle, beta), *Tnnc1 *(troponin C, cardiac/slow skeletal), and *Tnni1 *(troponin I, skeletal, slow 1) were represented. The profiles of Sox6 binding and Pol II binding for these genes are summarized in Additional file 3, Figure S2A-E. Except for *Tnnc1 *(*4.72 RPKM*) and *Tnni1 *(*3.52 RPKM*), Pol II binding levels of these genes were very low (Additional file 3, Figure S2A-E). It should be noted that Sox6 peaks were not detected for *Myh4 *(Additional file 3, Figure S2A) which encodes the fastest adult myosin isoform MyHC-IIb [2,3] nor for *Myh8 *(data not shown) which encodes the perinatal fast MyHC isoform [5]. This suggests that Sox6 is not directly involved in transcriptional regulation of the fastest MyHC isoforms expressed in fetal or adult skeletal muscle.

**Table 3:** Biological processes enriched among genes associated with Sox6 peaks

GO term	*P *value
GO:0015629~actin cytoskeleton	2.58E-013
GO:0008092~cytoskeletal protein binding	4.27E-013
GO:0005856~cytoskeleton	4.51E-012
GO:0003779~actin binding	2.93E-011
GO:0030016~myofibril	3.88E-008
GO:0043292~contractile fiber	7.80E-008
GO:0044430~cytoskeletal part	1.67E-007
GO:0030054~cell junction	6.40E-007
GO:0045944~positive regulation of transcription from RNA polymerase II promoter	1.40E-006
GO:0045893~positive regulation of transcription, DNA-dependent	2.93E-006
GO:0051254~positive regulation of RNA metabolic process	3.50E-006
GO:0007517~muscle organ development	5.48E-006
GO:0030017~sarcomere	5.82E-006
GO:0043228~non-membrane-bounded organelle	6.45E-006
GO:0043232~intracellular non-membrane-bounded organelle	6.45E-006
GO:0006357~regulation of transcription from RNA polymerase II promoter	7.81E-006
GO:0007010~cytoskeleton organization	8.98E-006
GO:0010628~positive regulation of gene expression	1.05E-005
GO:0045941~positive regulation of transcription	1.25E-005
GO:0045935~positive regulation of nucleobase, nucleoside, nucleotide and nucleic acid metabolic process	1.37E-005
GO:0044449~contractile fiber part	1.39E-005
GO:0010557~positive regulation of macromolecule biosynthetic process	1.59E-005
GO:0060537~muscle tissue development	1.77E-005
GO:0031328~positive regulation of cellular biosynthetic process	2.01E-005
GO:0009891~positive regulation of biosynthetic process	2.50E-005
GO:0007507~heart development	2.69E-005
GO:0048729~tissue morphogenesis	2.78E-005
GO:0051173~positive regulation of nitrogen compound metabolic process	2.88E-005
GO:0042692~muscle cell differentiation	3.41E-005
GO:0006936~muscle contraction	3.79E-005

The thirty most enriched Gene Ontology (GO) biological process terms are listed.

Another noticeable GO term category enriched in the genes associated with Sox6 peaks involved regulation of transcription (Table 3). For instance, Sox6 peaks were found in the vicinity or in the gene region of transcriptional regulators including (but not limited to) *Prox1, Sox6, Tead1*, *Tead4*, *Tcf4*, *Hdac9*, *Hdac11*, and *Nfatc3 *(Additional file 3, Figure S2F-M). These genes (except for *Hdac11*) are known to play a role in not only skeletal muscle development, but also heart development [12,13,38,42-49]. In spite of its high expression in skeletal muscle, the role of the class IV histone deacetylase Hdac11 [50] in muscle development is yet to be discovered [51].

*Prox1 *encodes a transcription factor expressed in slow muscle in zebrafish [47]. Though its role in mammalian skeletal muscle development is yet to be reported, we hypothesize that the Prox1 protein also plays a role in slow muscle fiber differentiation in mice. To support this, we have found that Prox1 mRNA is preferentially expressed in the slow soleus muscle compared to the EDL, TA, and gastrocnemius muscles in adult (Additional file 1, Figure S1B). Therefore, the Prox1 protein may play a role in slow fiber differentiation during muscle development as well as maintenance of slow muscle in adult. In the Sox6 gene region, two Sox6 peaks were detected in the fifth intron (Additional file 3, Figure S2G). Existence of Sox6 binding sites and very low levels of Pol II binding in the Sox6 gene region may suggest a self-regulatory mechanism of Sox6 transcription during skeletal muscle development, as has been recently reported for erythrocyte development [52].

We also examined whether *Tead1*, *Tead4, Tcf4*, *Hdac9 *and *Hdac11 *are differentially expressed between slow and fast muscles. We found that *Tead1, Tead4, Tcf4 *and *Hdac9 *were all expressed higher in the slow soleus muscle than the group of fast muscles, EDL, TA, and gastrocnemius (Additional file 1, Figure S1C-F). *Hdac11*, on the other hand, was expressed slightly higher in the fast muscles than soleus (Additional file 1, Figure S1G). These results suggest that Tead1, Tead4, Tcf4 and Hdac9 may also positively regulate slow fiber specific genes. The association of Sox6 peaks to these transcriptional regulatory genes suggests that Sox6 may be indirectly regulating muscle development through these key transcription regulators.

Sox6 binding to the genes described above was validated by ChIP-qPCR (Additional file 4, Figure S3).

### Muscle specific inactivation of Sox6 results in significant upregulation of slow fiber, cardiac, and developmental isoform genes in skeletal muscle

The observation that the majority (84%) of the genes associated with Sox6 binding sites show little or no Pol II binding (Figure 4) supports our hypothesis that a major function of Sox6 during myogenesis is transcriptional suppression. To further evaluate this hypothesis, we next analyzed mRNA expression of selected genes associated with Sox6 binding in Sox6 KO muscle. For this, we used MCK-Cre mice (harboring the Cre gene under the control of the muscle creatin kinase promoter) to assess the effect of Sox6 inactivation in skeletal muscle as well as in cardiac muscle [53,54], since many of the putative Sox6 target genes are also expressed in the heart.

**Figure 5:**
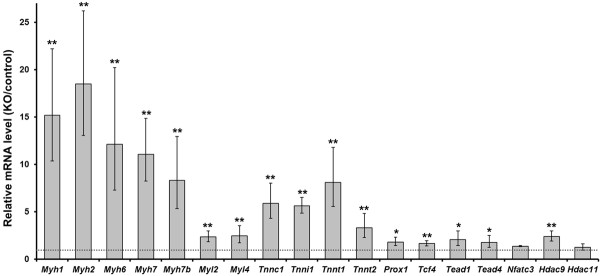
**Differences of expression levels of Sox6 target genes between control and Sox6 KO perinatal mice**. RT-qPCR was performed using total RNA from skeletal muscle of control (Sox6^f/f^) and Sox6 KO (Sox6^f/f^; MCK-Cre) newborn (postnatal day 1) mice. Expression levels in Sox6 KO mice were divided by those in control mice, and represented as mean ± SD (*n *= 3). The broken line corresponds to the expression ratio of 1, indicating equal expression level between the KO and control mice. (*) *P *< 0.05; (**) *P *< 0.005. Fiber type specific genes: *Myh1, Myh2, Myh7, My7b, Myl2 *(also expressed in the heart), *Tnnc1 *(also expressed in the heart), *Tnni1, Tnnt1*; cardiac isoforms: *Myh6, Tnnt2*; developmental isoform: *Myl4*; transcription factors: *Prox1, Tcf4, Tead1, Tead4, Nfatc3*; histone modification enzymes: *Hdac9, Hdac11*.

First, mRNA levels of the eighteen genes (eight fiber type-specific genes, two cardiac isoform genes, one developmental isoform gene and five transcription factors and two histone modification enzyme genes) were compared between control and Sox6 KO mice using newborn skeletal muscle (Figure 5). Sixteen genes out of the eighteen tested showed a significant increase in mRNA expression in the newborn Sox6 KO skeletal muscle (Figure 5). *Nfatc3 *and *Hdac11 *showed a tendency to be increased in Sox6 KO muscle, even though the difference was not statistically significant (Figure 5). These results indicate that Sox6 functions as a suppressor for these genes in developing muscle.

Next, we assessed mRNA expression of fifteen genes out of the eighteen tested above (*Myl4*, *Nfatc3*, and *Hdac11 *were excluded) as well as fast fiber specific genes, myogenic regulatory factors, and metabolism related genes in adult Sox6 KO muscle (Table 2). The slow fiber specific sarcomeric protein genes which had shown increased expression in Sox6 KO newborn muscle (*Myh7*, *Myl2*, *Tnnc1*, *Tnni1*, and *Tnnt1*) displayed an even greater fold increase in mRNA expression in adult Sox6 KO muscles compared to control (Table 2). Among the four muscle groups tested, the TA and EDL Sox6 KO muscles showed the most dramatic increase in slow fiber specific gene expression, the soleus exhibiting the least fold increase (Table 2), again likely reflecting the lower Sox6 expression in the soleus than the fast muscles TA, EDL, and gastrocnemius (Additional file 1, Figure S1A). The fast fiber specific genes (*Myh4*, *Tnni2*, and *Tnnt3*) exhibited a significant decrease in their mRNA expression in Sox6 KO muscles (Table 2). *Myh1 *(IIx/d) and *Myh2 *(IIa), were either increased or decreased in different Sox6 KO muscle groups (Table 2), which may reflect the fluid nature of MyHCs IIa and IIx/d's expression in adult skeletal muscle [2]. These two MyHC isoforms are intermediates between MyHC-β and MyHC-IIb when fiber type shift occurs in skeletal muscle. Therefore, they could be more sensitive to the timing and level of the Sox6 gene inactivation, leading to varied expression in the individual Sox6 KO muscles. Upregulation of the cardiac isoform genes, *Myh6 *and *Tnnt2*, was also observed in the adult Sox6 KO muscle (Table 2).

The significant upregulation in the slow fiber and cardiac isoform gene expression in adult Sox6 KO skeletal muscle likely suggests that inactivation of the Sox6 gene early in myogenic development inhibited the postnatal maturation of the skeletal muscle. Postnatal development of skeletal muscle is characterized by the progressive decline of slow fiber specific gene expression in fast muscles [6,7,55]. As a result, control EDL and TA muscles express only a trace amount of the MyHC-β protein [6,56,57]. The extreme upregulation of the slow fiber specific genes such as *Myh7*, *Tnnc1*, and *Tnnt1 *in the Sox6 KO fast muscles may reflect their suspended postnatal maturation. This delayed maturation hypothesis is supported by the observation that the embryonic isoform acetylcholine receptor (Ach-R) γ (*Chrng*) is expressed at a higher level than the adult isoform Ach-R ε (Chrne) in Sox6 KO muscles (Table 2). During postnatal maturation of skeletal muscle, Ach-R γ is replaced by the adult isoform Ach-R ε [58]. In the adult Sox6 KO muscles, silencing of *Chrng *was not seen and *Chrne *expression did not reach to the control level (Table 2). Since we have located one Sox6 peak in the *Chrng *promoter region (approximately 135 bp upstream of the TSS), Sox6 may be directly suppressing transcription of *Chrng *during normal skeletal muscle development.

### Transcriptional regulatory genes associated with Sox6 peaks are upregulated in Sox6 KO adult muscle

In addition to the sarcomeric protein genes, mRNA levels of some of the transcriptional regulatory genes associated with Sox6 peaks were upregulated in the Sox6 adult KO skeletal muscles. *Prox1 *expression was significantly increased in Sox6 KO muscles, with the highest fold increase in the TA and EDL, followed by the gastrocnemius (Table 2). It should be noted that *Prox1 *expression is highest in the soleus in the adult control (Sox6^f/f^) muscles (Additional file 1, Figure S1B). These observations suggest that *Prox1 *may play a role for sustaining slow fiber specific gene expression in adult muscle. *Tead4 *and *Hdac9 *also showed a slight increase in their expression in the Sox6 KO TA, EDL, and gastrocnemius muscles (Table 2). Expression of *Tcf4 *and *Tead1*, on the other hand, showed no clear difference between Sox6 KO and control adult muscles (Table 2), in spite of their higher expression in the Sox6 KO newborn muscle (Figure 5). This result suggests that Sox6 may regulate transcription of *Tcf4 *and *Tead1 *in developing muscle, but this regulation may not be maintained through adult.

Since it has previously been reported that MyoD and Myogenin are differentially expressed between slow and fast muscles (MyoD higher in fast than slow; Myogenin higher in slow than fast) [59,60], we also examined mRNA expression of these genes in Sox6 KO muscle. As shown in Table 2, there was no discernable change in MyoD mRNA expression, whereas there was a small increase in Myogenin mRNA expression in Sox6 KO muscles. An increase in Myogenin expression in Sox6 KO muscle suggests that Myogenin may play some role in maintaining slow fiber phenotype in the adult skeletal muscle as previously proposed [60].

### The level of transcriptional upregulation of metabolism related genes is less than that of slow fiber sarcomere protein genes in Sox6 KO muscle

**Figure 6:**
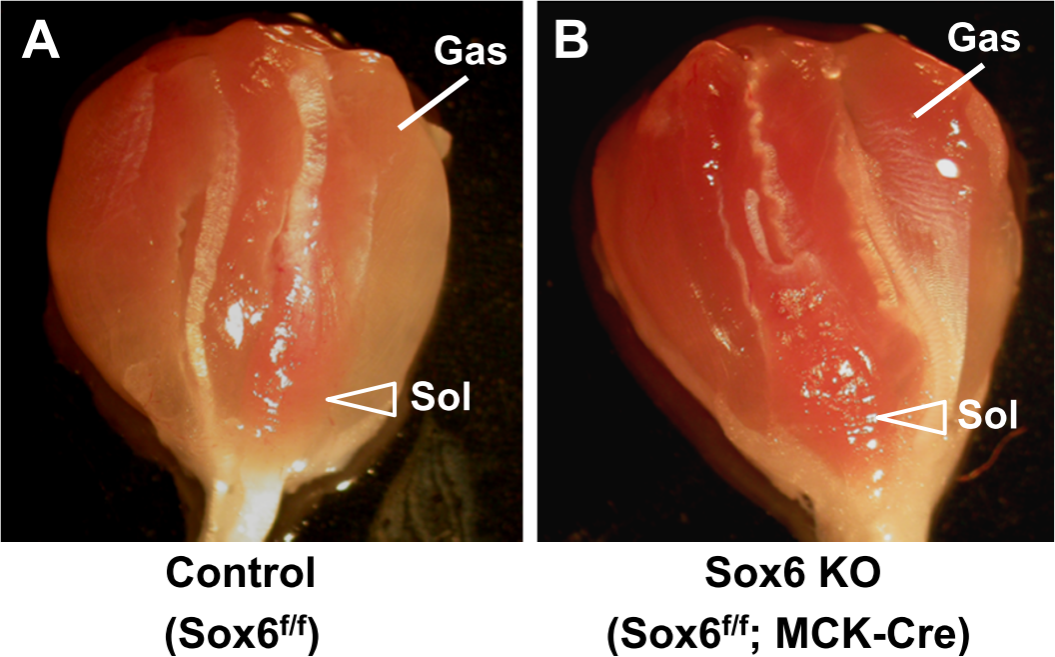
**Morphological difference of skeletal muscle between control and Sox6 KO mice**. Dissected gastrocnemius/soleus muscles from three month old mice are shown. A. control (Sox6^f/f^) muscle. B. Sox6 KO (Sox6^f/f^; MCK-Cre) muscle.

Since a close coupling between the slow fiber gene program and the oxidative metabolism gene program in adult skeletal muscle has been reported [1,3], we also examined mRNA expression of the genes whose high expression is correlated with the oxidative state of skeletal muscle metabolism in Sox6 KO muscle (myoglobin, Sdha and Ppargc1a). In MCK-Cre induced Sox6 KO muscle, mRNA levels of *Ppargc1a *and *Sdha *were, in general, lower than control (Table 2). These results replicated the data obtained using Myf5-Cre induced Sox6 KO muscle (Table 1). Myoglobin expression showed a slight increase in the Sox6 KO TA, EDL, and gastrocnemius (Table 2). When the color of gastrocnemius and soleus muscles was visually inspected, the characteristic color difference between the two muscles in control muscle (soleus being redder than gastrocnemius) was less clear in Sox6 KO muscle, because the Sox6 KO gastrocnemius exhibited an increase in redness in its color (Figure 6). This may reflect a small, but consistent increase in myoglobin expression in the Sox6 KO gastrocnemius muscle (Table 2). The more red muscle in Sox6 KO muscle has been also reported by Quiat et al. [34]. Interestingly, both Quiat et al. and our current report observed reduced expression in *Ppargc1a *in Sox6 KO muscle, which may suggest that there could be a pathway independent of *Ppargc1a *regulating myoglobin expression in Sox6 KO muscle. An alternative explanation for the increased redness in the Sox6 KO muscle could be a change in capillary density. In the Sox6 peak associated RefSeq genes, GO terms related to vasculature development and angiogenesis were also enriched (*P *< 2 × 10^-3^) (Additional file 5, Table S2). Thus, the increased capillary density could be the cause of more red color of the Sox6 KO gastrocnemius.

### Fetal isoform gene expression is upregulated in the Sox6 KO heart

Since the two cardiac MyHC isoform genes, α and β (*Myh6 *and *Myh7*), were associated with Sox6 binding (Additional file 3, Figure S2B) and their expression was upregulated in Sox6 KO skeletal muscle (Figure 5, Table 2), we next examined their expression in the Sox6 KO heart. In the mouse heart, expression of MyHC-α and MyHC-β is developmentally regulated. MyHC-β is the fetal isoform in the heart and is replaced by the adult isoform MyHC-α within the first week after birth [61]. As summarized in Table 4 it appears that this isoform transition, fetal to adult, is incomplete in the Sox6 KO myocardium. In the Sox6 KO heart, MyHC-β expression was sustained at an equal to a slightly higher level than control heart, whereas MyHC-α expression decreased to approximately the half of the control level (Table 4). To test if this is a developmental defect in the postnatal heart, we also examined expression of the developmentally regulated skeletal α-actin gene, which is expressed in the fetal heart and silenced later in adult [62]. Indeed, skeletal α-actin mRNA expression was consistently higher in the Sox6 KO heart (Table 4), suggesting that the Sox6 KO heart is developmentally more immature than the control heart. Interestingly, the expression of *Ppargc1 *was also lower in the Sox6 KO heart (Table 4). Since Ppargc1 plays an important role in maturation of the metabolic state and mitochondrial biogenesis in the postnatal heart [63-65], this result suggests that the loss of Sox6 caused a delay in the postnatal maturation of the heart, thus Sox6 may also be necessary for the functional maturation of cardiac muscle.

**Table 4:** Fold change in mRNA levels in the Sox6 KO heart compared to control

	Heart		
	
Mouse ID#	1	2	3
Sox6	0.23	0.16	0.30
Myh6	0.67	0.61	0.51
Myh7	3.40	1.78	1.25
Acta1 (sk-actin)	4.28	4.36	2.55
Ppargc1a	0.52	0.57	0.51

Sox6 was inactivated using MCK-Cre mice. Mouse 1 and mouse 2 are two month-old, and mouse 3 is three month-old. Control expression level = 1.00.

### Nfatc3 protein expression is highly upregulated in Sox6 null myotube cultures

**Figure 7:**
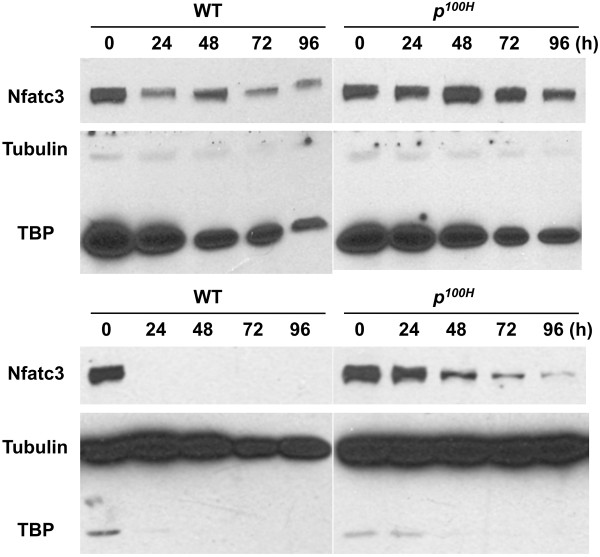
**Nfatc3 protein levels are increased in both the nucleus and cytoplasm of Sox6-null myotubes**. Wild type (WT) and Sox6-null (*p^100H^*) fetal primary myoblasts or myotubes were harvested at 0, 24, 48, 72, and 96 h after switching to DM, and nuclear and cytoplasmic protein was extracted for Nfatc3 Western blotting. TATA binding protein (TBP) and tubulin were used as loading controls for nuclear and cytoplasmic fractions, respectively. Upper panels: nuclear fractions. Lower panels: cytoplasmic fractions.

It has been reported that Nfatc3 stimulates myogenic differentiation both in vivo and in vitro [43,45]; however, its implication in muscle fiber type specification has not been noted. Calcineurin-directed dephosphorylation of NFAT factors results in their nuclear localization and transcriptional activation of their target genes [66]. We have located one Sox6 peak in the last intron of *Nfatc3 *(Additional file 3, Figure S2J). As shown in Figure 5, Sox6 KO newborn skeletal muscle showed a small increase (not statistically significant) in *Nfatc3 *mRNA expression. To assess whether the Nfatc3 activity increases in Sox6 null myotubes, we examined sub-cellular localization the Nfatc3 protein using Western blot. We took advantage of the Sox6 null mouse (*p^100H^*-Sox6 null mutant allele) in our laboratory to obtain a pure population of Sox6 null myotubes [12,13,26]. Fetal myoblasts were prepared from E18.5 *p^100H^*-Sox6 null and wild type littermates and were differentiated in differentiation medium (DM). In undifferentiated myoblast cultures, the amount of the nuclear as well as cytoplasmic Nfatc3 protein was comparable between Sox6 null and wild type (Figure 7). Once myotube differentiation was induced, in wild type cultures, the Nfatc3 protein was detected only in the nuclear fraction, whereas in the *p^100H ^*cultures, a continuous presence of the cytoplasmic Nfatc3 protein and a higher level of the nuclear Nfatc3 protein (compared to wild type) were observed (Figure 7). We have previously reported that Sox6 expression is significantly increased upon induction of myotube differentiation [13]. Therefore, these results suggest that a higher level of Sox6 expression in wild type myotubes likely suppressed new synthesis of Nfatc3, while the absence of Sox6 in *p^100H ^*myotubes allowed continuous Nfatc3 synthesis. These results suggest that Nfatc3 activity is upregulated in the Sox6 null myotubes which show a higher level of slow fiber specific gene expression.

### Functional analysis of the Sox6 binding sites

**Figure 8:**
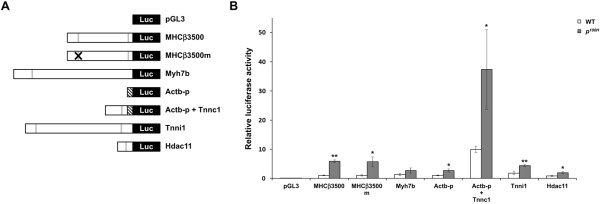
**Differences of reporter activities between wild type and Sox6-null primary myotubes**. A. Schematic representations of the firefly luciferase vectors constructed to test the function of Sox6 binding sequences. Black boxes indicate the firefly luciferase gene and shaded boxes indicate the chicken β-actin promoter (Actb-p). Open boxes indicate the upstream sequences of the wild type MyHC-β gene (MHCβ3500) or its mutated version (MHCβ3500 m; cross indicates mutation), *Myh7b*, *Tnni1*, and *Hdac11*, or the first intron sequence of *Tnnc1*. Approximate positions of Sox6 binding sites are indicated by gray lines. B. The reporter constructs shown in A were cotransfected with a Renilla luciferase vector into wild type (WT) and Sox6-null (*p^100H^*) primary myoblasts. After differentiation into myotubes, both luciferase activities were measured and normalized with Renilla luciferase activity. Data were further normalized to WT MHCβ3500 value (i.e. MHCβ3500 in WT = 1.0), and represented as mean ± SD (*n *= 3). (*) *P *< 0.05; (**) *P *< 0.005.

In order to characterize the functional nature of the Sox6 binding sites in transcriptional regulation, we next performed reporter gene assays. We chose five Sox6 peak-associated genes, *Myh7 *(MyHC-β), *Myh7b*, *Tnnc1, Tnni1*and *Hdac11*, in which Sox6 binding was validated by ChIP-qPCR (Additional file 4, Figure S3). All of these Sox6 peaks tested contained a Sox consensus motif. Firefly luciferase vectors containing each of the following sequences, ~3.5 kb *Myh*7 5'-upstream sequence (two Sox6 peaks; MHC3500), ~6 kb *Myh7b *5'-upstearm sequence (one Sox6 peak), ~1.3 kb of the *Tnnc1 *first intron (one Sox6 peak), ~5.2 kb *Tnni1 *5'-upstream region (two Sox6 peaks), and ~1 kb *Hdac11 *5'-upstream sequence (one Sox6 peak) were generated (Figure 8A; see Additional file 3, Figure S2B-E and S2M for the location of Sox6 peaks). It should be noted that the proximal Sox6 peak in the *Tnni1 *5'-upstream region (approximately -800 bp from TSS) overlapped with the previously reported slow upstream regulatory element (SURE) containing an enhancer element [67].

To assess whether these Sox6 binding sites function as a negative or positive regulatory element, the luciferase reporter gene constructs described above were transiently transfected to *p^100H^*-Sox6 null and wild type myoblasts, differentiated in DM for 48 hours, after which firefly luciferase activities were compared between the Sox6 null and wild type myotube cultures. If these Sox6 binding sequences function as negative regulatory regions, it is expected that the luciferase activity would be higher in *p^100H ^*myotube cultures in which no functional Sox6 protein is produced. As summarized in Figure 8B, four out of the five sequences tested drove a higher firefly luciferase activity in Sox6 null myotube cultures compared to wild type, indicating that these Sox6 binding sites function as negative regulatory sequences. The *Myh7b *5'-sequence did not drive a statistically higher luciferase activity in Sox6 null myotube cultures (Figure 8B). Since the endogenous *Myh7b *expression was higher in Sox6 KO muscle (Figure 5), it is possible that the in vitro culture may not be the best approach to assess the effect of the *Myh7b *Sox6 biding regions. Intriguingly, Bell et al. have shown that Sox6 protein overexpression in C2C12 cells could suppress transcription from the 1 kb *Myh7b *5'-upstream sequence [68]. Therefore, there could be a Sox6 binding site not detected in our ChIP-seq analysis which may still be functioning as a negative regulatory element in a different context.

We have previously shown that the proximal Sox6 binding site (-200 bp from the *Myh7 *TSS) functions as a negative regulatory element in reporter gene assays [13]. In the present report, we have identified an additional distal Sox6 binding site (-2900 bp from TSS) which overlaps with a known muscle enhancer element [69,70]. To delineate the two Sox6 binding sites in the *Myh7 *5'-upstrem region (see Additional file 3, Figure S2B for the peak locations), the distal Sox consensus sequence (-2,900 bp) was mutated in MHCβ3500 (designated as MHC β3500 m) (Figure 8A). As shown in Figure 8B, the loss of the distal Sox motif did not affect the luciferase activity in either Sox6 null or wild type myotubes. This result suggests that the distal Sox motif has little effect on transcriptional suppression from the 3.5 kb *Myh7 *5'-region in transient assays, and therefore, at least in the current in vitro assay conditions, the proximal Sox6 binding site is sufficient to suppress the transcription driven by the 3.5 kb *Myh7 *5'-upstream region.

The function of the Sox6 binding site in the *Tnnc1 *first intron was determined using a hybrid luciferase reporter construct whose transcription is driven by the chicken β-actin promoter. The *Tnnc1 *first intron contains an enhancer element which was previously identified using C2C12 and Sol8 skeletal muscle cell lines [41]. The presence of this intron alone significantly increased luciferase activity in wild type myotubes (Actb-p vs. Actb-p+Tnnc1, p < 0.0001), confirming the enhancer activity (Figure 8B). The luciferase activity of the construct, Actb-p+Tnnc1, in Sox6 null myotubes was significantly higher than wild type, indicating that Sox6 binding hindered the enhancer activity in this intron (Figure 8B). Unexpectedly, the construct containing only the chicken β-actin promoter exhibited a small but statistically significant increase in luciferase activity in Sox6 null myotube cultures compared to wild type (Figure 8B). This was likely caused by the fortuitous presence of a couple of Sox motif sequences in the chicken β-actin promoter and intron sequences in the vector (data not shown), which could have functioned as a weak silencer element. The 5'-upstream sequences of both *Tnni1 *and *Hdac11 *showed a moderate but statistically significant increase in luciferase activity in Sox6 null myotubes (Figure 8).

## Discussion

In order to understand how Sox6 regulates muscle differentiation at the molecular level, we have performed ChIP-seq analysis to identify Sox6 targets in skeletal myotubes and extended the characterization of the Sox6 null muscle phenotype using muscle specific Sox6 inactivation. Among the 867 Refseq genes found to be associated with Sox6 peaks, the overrepresented GO terms included muscle structure and function, skeletal muscle and heart development, as well as transcriptional regulation. In a concurrently conducted Pol II ChIP-seq analysis, we found that the majority of the Sox6 peak-associated genes exhibited little to no recognizable binding peaks, suggesting that Sox6 mainly functions as a transcription suppressor in developing muscle.

How does Sox6 suppress its target genes? Based on evidence from this and other labs, we can speculate on two possible mechanisms (1 and 2) and, based on evidence accumulated in this report we also demonstrate two other likely mechanisms (3 and 4): (1) Sox6 may fine-tune the transcription of the genes that have been marked by MyoD binding, (2) Sox6 may modulate transcription of its target genes in concert with Tead and Runx factors, (3) Sox6 suppresses transcription by hindering the muscle-specific enhancer activity, and (4) Sox6 also indirectly influences downstream gene expression by regulating the expression of other transcription factors and chromatin modifying enzymes. Below, we will discuss each of these proposed mechanisms in more detail.

MyoD is one of the myogenic regulatory factors and defines the myogenic lineage during development [71,72]. In myotubes, MyoD binding events are frequent (~26,000 peaks with a higher cut off, ~60,000 peaks with a lower cut off) and are associated with histone H4 acetylation (H4Ac) [36], which is a marker of an active chromatin state [73]. We found that 96% of the Sox6 peaks in fetal myotubes overlapped with, or were in the close vicinity to (within 50 bp), the reported MyoD peaks [34]. The E-box motifs in the Sox6 peak regions we found were enriched for the CAGCTG E-box sequence (Figure 3). Previously, it has been shown that this motif is represented in the peaks more strongly bound in C2C12 myotubes compared to myoblasts, indicating that this E-box motif is mainly associated with the genes regulating muscle differentiation [36]. Taking this observation together with ours, we speculate that MyoD binding in the myotube would change the chromatin environment in such a way as to allow the approach of additional transcriptional regulators by recruiting the chromatin modifying enzymes [74], thus allowing the fine-tuning of muscle specific gene expression necessary for the formation of mature skeletal muscle. Sox6 could be one of these additional transcriptional regulators and specify fiber type characteristics during muscle terminal differentiation.

We have previously reported that Sox6 interferes with a MCAT enhancer located in close proximity to the Sox consensus motif in *Myh7*, causing suppression of *Myh7 *transcription [13]. Tead/MCAT motifs are frequently found in enhancer or promoter regions of muscle specific genes and it has been demonstrated that binding of TEF-1/Tead1 to the MCAT motifs activates transcription of these muscle-specific genes [38,75]. In our analysis of the 1,066 Sox6 peaks, we found 203 MCAT motifs. This suggests that the mechanism of Myh7 transcriptional suppression by Sox6 (possibly via physical interference) we reported earlier may be a common mechanism Sox6 uses to suppress genes whose transcription is activated via Tead/MCAT motifs. Our analysis also revealed 559 Runx motifs in the 1,066 Sox6 peaks. Currently, the roles of Runx motif binding factors (Runx-1, -2, and 3) in muscle development are not well known, though there are reports showing that Runx1 plays a role in skeletal muscle differentiation [37,76,77]. In adult skeletal muscle, Runx1 expression is induced by denervation [77], and muscle-specific Runx1 inactivation leads to accelerated muscle wasting in denervated muscle [76]. In an earlier stage of muscle differentiation, it has been reported that Runx1 directly interacts with MyoD preferentially in proliferating myoblasts to inhibit terminal differentiation of skeletal muscle [37]. The authors showed that the Runx1/CBFβ complex recruits suppressive chromatin modifying enzymes (e.g. HDACs), thus inactivating transcription of the MyoD target genes that are necessary for the cell cycle exit and differentiation [37]. Since the Runx proteins have been shown to function as transcriptional suppressors or activators in different circumstances [78] (similar to Sox6), the transcriptional outcome of the possible interaction between the Sox6 and Runx proteins needs further investigation.

As demonstrated in the Results section, the Sox6 binding sites in the *Tnnc1 *first intron and the *Tnni*1 5'-upstream region both effectively reduced the activity of the enhancer elements (Figure 8). The molecular mechanisms by which Sox6 overrides muscle enhancers is currently under investigation; however, the skeletal muscle MyHC gene clusters may help shed light on this role of Sox6. In the six MyHC isoform genes clustered on the mouse chromosome 11 [*Myh3 *(emb), *Myh2 *(IIa), *Myh1 *(IIx/d), *Myh4 *(IIb), *Myh8 *(peri), *Myh13 *(eo)] [74], only the *Myh4 *and *Myh8 *genes were not associated with Sox6 peaks (a Sox6 peak was detected in the 5'-upstream region of *Myh13 *in one of the two ChIP-seq data sets; data not shown). Therefore, Sox6 may be involved in sequential expression of the MyHC loci, possibly in collaboration with an enhancer element similar to the locus control region (LCR) reported for the globin gene cluster [79]. This is an appealing hypothesis, because it has been shown that Sox6 (acting as a transcriptional suppressor) regulates sequential expression of the β-globin genes during erythrogenesis [80] in concert with BCL11A which binds to the globin gene LCR [81]. There have been reports on transcription factories that unite transcriptionally active genes on separate chromosome regions for coordinated transcription [82]. It is possible that association of Sox6 with its target sequences inhibits transcriptional initiation by Pol II, thus causing dissociation of Sox6 target genes from transcription factories.

We demonstrated that expression of *Tead1*, *Tead4*, *Hdac9*, and *Prox1 *was upregulated in Sox6 KO skeletal muscle (Figure 5), suggesting that Sox6 is a suppressor of these transcriptional regulatory genes. Tead1 (TEF-1) and Tead4 (RTEF-1) are highly expressed in muscle tissues and have been reported to activate muscle specific gene transcription [83-85]. Hdac9 is a class IIa HDAC [86] and functions as a mediator of motor neuron input to skeletal muscle [87]. Prox1 is expressed in slow muscle in zebrafish [47]. Since *Prox1 *is preferentially expressed in slow fiber muscle in control mice (Additional file 1, Figure S1B) and Sox6 inactivation caused a sizable increase in *Prox1 *mRNA expression in Sox6 KO muscle, we propose that Prox1 also plays a role in regulation of slow muscle fiber specific gene expression in mice. This observation presents further evidence of evolutionary conservation in the mechanisms regulating muscle fiber type differentiation in vertebrates [19,88]. Since there are more transcriptional regulator genes that are closely associated with Sox6 peaks, which we did not have space to discuss in this report, it is likely that Sox6 is part of the transcriptional networks that shape the characteristics of both muscle development and mature muscle functions.

The most striking phenotype of Sox6 null skeletal muscle is the dramatic increase in the expression of multiple slow fiber specific genes. This observation originally led us to hypothesize that Sox6 functions as a transcriptional suppressor of slow fiber specific genes [12,13]. In this report, we expanded the gene expression profiling of Sox6 KO skeletal muscle by including cardiac and embryonic muscle isoform genes. Cardiac isoforms *Myh6 *and *Tnnt2*, as well as embryonic isoforms *Myl4 *and *Chrng*, were upregulated in the Sox6 KO muscle (Figure 5, Table 2). It has been reported that *Tnnt2 *is upregulated in regenerating dystrophic muscle [89]. *Myh6 *is expressed in specialized craniofacial muscle, such as jaw and extraocular muscle, but not in limb or other body muscle [90,91]. These observations suggest that Sox6 may play a role in not only determining fiber types, but also defining developmental maturity and highly specialized functions of skeletal muscle.

In Sox6 KO muscle, a significant decrease in fast fiber specific gene expression was also observed. This Sox6 KO phenotype could be a secondary effect of the increased slow fiber gene products, or could be regulated indirectly by Sox6. Since we did not find Sox6 peaks associated with fast fiber specific genes, both mechanisms are equally plausible. With regard to indirect regulation, a few possible mechanisms can be hypothesized. For example, expression of the transcription factors Six1 and Six4, activators of fast fiber specific gene expression [29,92], could be indirectly suppressed in Sox6 KO muscle during development. Alternatively, downregulation of fast fiber specific genes in Sox6 KO muscle could be caused by changes in microRNA expression. MicroRNAs are known to function as posttranscriptional regulators of gene expression [93]. A recent report indicates that microRNAs suppress target gene expression predominantly through mRNA degradation [94], thus, it is plausible to postulate that an increase in microRNAs targeting fast fiber specific genes in Sox6 KO muscle leads to reduced fast fiber specific gene mRNA levels. As described above, we found Sox6 binding peaks associated with *Myh6 *and *Myh7 *(Additional file 3, Figure S2B). In the intron sequences of *Myh6 *and *Myh7*, miR-208a and miR-208b are encoded, respectively [95]. It has been reported that miR-208 suppresses expression of THRAP1, which promotes fast fiber specific gene expression [96]. The increased transcription of *Myh6 *and *Myh7 *in Sox6 KO muscle, therefore, could lead to upregulation of miR-208, which in turn, suppress fast fiber specific gene expression. However, the actual situation is likely to be more complex. It should be noted that miR-208, along with miR-499, also targets the 3'-UTR region of *Sox6 *[68,97,98]. MiR-499 is encoded in the intron of *Myh7b *[95,99], which has a Sox6 binding site in its 5'-upstream region (Additional file 3, Figure S2C). Since *Myh6*, *Myh7*, and *Myh7b *are all negatively regulated by Sox6 (Figure 5), these data suggest that Sox6 and these miRNAs constitute two-way feedback loops.

**Figure 9:**
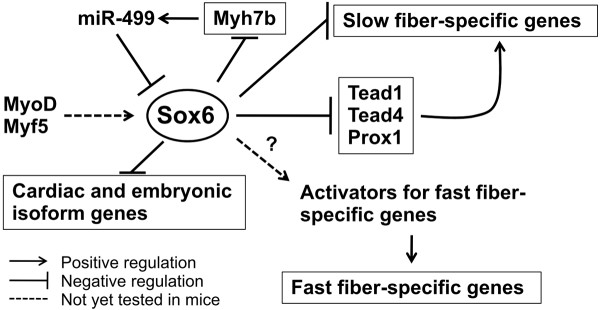
**Summary of the present work concerning the fiber type specification under the control of Sox6**. It has been shown that in zebrafish, MyoD and Myf5 are necessary to activate Sox6 gene expression in muscle [100]. This muscle-specific Sox6 activation mechanism has not been tested in mice yet, but since Sox6 upregulation coincides with upregulation of these myogenic regulatory factors during muscle differentiation, it is very likely that this mechanism is shared in mice also (see text). Once expressed in muscle, Sox6 directly suppresses transcription of slow fiber specific, cardiac and embryonic isoform genes during muscle development. In addition to these structural protein genes, Sox6 suppresses expression of the transcription factors which have been shown to activate slow fiber specific genes, Tead1, Tead4, and Prox1. By unknown mechanisms, fast fiber-specific gene expression in Sox6 KO skeletal muscle is dramatically reduced. Since Sox6 is preferentially expressed in fast-twitch fiber rich muscles, it is possible that Sox6 indirectly stimulates the fast fiber-specific gene program. This idea awaits future investigation. Sox6 activity in slow fibers is suppressed by miR-499 which is encoded in an intron of the Myh7b gene. We have shown that Sox6, in turn, suppresses Myh7b transcription. This negative feedback loop might be important for fiber type switching during muscle development as well as in adult muscle.

Figure 9 summarizes both our current results and the reported regulatory mechanisms for Sox6 expression. A recent report on the regulation of Sox6 expression in zebrafish skeletal muscle has demonstrated that Sox6 transcription is positively regulated by MyoD and Myf5, and repression of Sox6 activity in slow fibers is maintained by miR-499 which targets the Sox6 3'-UTR [100]. We have reported that Sox6 transcription is upregulated when myotube differentiation is induced [13], therefore, MyoD and Myf5 might also be activating Sox6 transcription during mammalian muscle development. Since MyoD is preferentially expressed in fast fibers in adult mice [59,60], it may sustain the higher level of Sox6 expression in adult fast fiber muscles reported here as well as by Quiat et al. [34]. Although the negative regulation of Sox6 by miR-499 has been already reported in mice [68,97,98], how suppression of Sox6 expression in slow fibers is initiated is not yet understood. Alternatively, it is also possible that Sox6 expression is activated when fast-twitch myotubes emerge during fetal muscle development [101]. Since fiber type-specific gene expression in mammalian skeletal muscle during development as well as in adult life is very fluid [2-4], how Sox6 expression is regulated will be an increasingly important question as we try to understand how muscle fiber type is initially specified, maintained and changed in reseponse to the external signaling.

## Conclusions

We have shown that: (1) Sox6 directly suppresses the transcription of slow fiber-specific, cardiac, and embryonic isoform genes through binding to the transcriptional regulatory regions, (2) Sox6 regulates expression of transcriptional regulators critical for muscle development, therefore, extending its effect on muscle development by cross-talking with other regulatory pathways, (3) Loss of Sox6 in skeletal muscle results in a significant increase in expression of slow fiber-specific, cardiac, and embryonic isoform genes which are associated with Sox6 binding peaks, accompanied by a decreased in fast fiber-specific gene expression, and (4) Loss of Sox6 in cardiac muscle results in increased expression of fetal isoform genes in the adult heart, which suggests that Sox6 is required for the postnatal maturation of cardiac muscle as well. Since the Sox6 KO phenotypes reported here have relevance to muscle degenerative diseases [102-105] as well as heart failure [106], uncovering the many functions of Sox6 in muscle development will likely contribute to the understanding of mechanisms of human muscular diseases.

## Methods

### Cell culture

Isolation, culture, and induction of myotube differentiation in differentiation medium (DM) of fetal myoblasts isolated from mouse E18.5 limb were described previously [13].

### ChIP-seq

ChIP experiments were performed using the Imprint Chromatin Immunoprecipitation Kit (Sigma-Aldrich) according to the manufacturer's instructions. Primary myotubes differentiated in DM for 48 h were washed with phosphate-buffered saline with 1 mM MgCl_2 _(PBS-Mg) once, and then fixed with 2 mM disuccinimidyl glutarate (DSG; Thermo Fisher Scientific) for 45 min at room temperature as described by [107]. Cells were washed with PBS-Mg twice, and fixed further using 1% formaldehyde for 10 min at room temperature. Antibodies used were rabbit polyclonal antibody to Sox6 (ab30455, Abcam), mouse monoclonal antibody to RNA Polymerase II (Pol II) CTD repeat YSPTSPS (4H8) (ab5408, Abcam), and normal mouse IgG from the ChIP kit. ChIP-seq library was prepared as described previously [108]. Briefly, the immunoprecipitated material was end-repaired, A-tailed, ligated to the sequencing adapters, amplified by 18-cycles of PCR and size selected (300-600 bp) followed by single end sequencing on an Illumina Genome Analyzer II by the DNA Technologies Core Facility at University of California, Davis http://dnatech.genomecenter.ucdavis.edu/. ChIP-seq data are available at Gene Expression Omnibus (accession number GSE32627).

### ChIP-seq data analysis

ChIP-seq reads and input sample reads were aligned using Bowtie (version 0.12.5) [109] with default parameters to the mouse NCBI Build 37 genome assembly. We obtained 3 and 1.5 million uniquely mapped reads from two independent Sox6 ChIP experiments, and 1.5 and 2.8 million uniquely mapped reads from two independent Pol II ChIP experiments, respectively. Peak calling was performed using SISSRs (version 1.4) [110] with the following parameters. -s (genome size): 2,716,965,481 bp, -F (average length of DNA fragments): 450 bp, -b (background file): input DNA data file of each ChIP experiment. Common peaks from the two Sox6 data sets were identified using ChIP-Seq Tool Set (version 1.0) [111]. Corresponding peaks within 50 bp were considered as overlapping. Peak annotation was carried out using PeakAnalyzer [112] with the default mouse mm9 annotation file. ChIP-seq data was visualized on the UCSC Genome Browser [113]. Motif discovery was conducted using MEME (version 4.5.0) [35] with default parameters, followed by comparison against three motif databases (JASPAR, TRANSFAC, and UNIPROBE) using TOMTOM (version 4.5.0) [114]. Gene ontology (GO) analysis was performed using the Database for Annotation, Visualization and Integrated Discovery (DAVID; http://david.abcc.ncifcrf.gov/) [115,116]. Pol II binding was represented as RPKM (reads per kilobase of RefSeq gene region per million mapped reads) based on read (tag) numbers in peak regions. We used the 2.8 million read data for calculation and visualization to maximize accuracy of result. When there are multiple RefSeq gene models per gene, the longest gene model was used for the calculations. When Pol II peaks were extended to extragenic regions, the length of extended regions was added to that of RefSeq gene models.

### qPCR

All measurements were conducted with ABI Prism 7900 HT Sequence Detection System (Applied Biosystems). ChIP-qPCR was performed using Maxima SYBR Green/ROX qPCR Master Mix (2X) (Fermentas) and specific primers listed in Additional file 6, Table S3. Single products were confirmed by dissociation curve analysis. Results were normalized to input, and fold enrichment was calculated by normalizing to enrichment at a negative control region (intergenic). RT-qPCR was performed using TaqMan Gene Expression Assays (Applied Biosystems). Total RNA was extracted with TRIzol reagent (Invitrogen). Following DNase treatment with DNA-free Kit (Ambion), cDNA was synthesized using High Capacity cDNA Reverse Transcription Kits (Applied Biosystems) with SUPERase-In (Ambion).

TaqMan probes used are provided in Additional file 7, Table S4. Results were normalized to β-actin (Actb) transcript level. All statistical analyses were performed using the two-tailed Student's t-tests. Relative mRNA levels against β-actin are shown in Additional files 8 and 9, Figures S4 and S5.

### Western blotting

Nuclear and cytoplasmic fractionation of primary myoblasts and myotubes was carried out using NE-PER Nuclear and Cytoplasmic Extraction Reagents (Thermo Fisher Scientific). A total of 30 μg of each protein sample was loaded on 7.5% SDS-polyacrylamide gel electrophoresis and transferred to a nitrocellulose membrane. The blot was incubated with anti-Nfatc3 mouse monoclonal antibody (sc-8405; Santa Cruz Biotechnology) at 1:100, and the signal was detected by Pierce ECL Western Blotting Substrate (Thermo Fisher Scientific). To estimate the amount of protein loaded in each lane, the same blot was stripped and then incubated with anti-TATA binding protein (TBP) mouse monoclonal antibody (ab818; Abcam) or anti α-Tubulin mouse monoclonal antibody (sc-8035; Santa Cruz Biotechnology) at 1:1000.

### Plasmid construction

A firefly luciferase expression vector driven by the MyHC-β promoter (MHCβ3500, which contains 3,500 bp of the 5' upstream sequence of the rat MyHC-β gene) was kindly provided by Dr. Baldwin at University of California, Irvine [117,118]. Using this vector as a template, a Sox motif in the distal Sox6 binding region found by our ChIP-seq experiments (approx. -2.9 kb in mouse) was mutated (TACAAAG to TCAGAAG) by an inverse PCR method [119] using KAPA HiFi HotStart DNA Polymerase (KAPA Biosystems) to generate MHCβ3500 m ("m" stands for mutation). Firefly luciferase expression vectors driven by the upstream regions of Myh7b, Tnni1, and Hdac11 genes (~ 6.0 kb, ~5.2 kb, and ~1.0 kb, respectively) were generated by inserting restriction enzyme-digested PCR products into appropriate restriction sites of pGL3-basic vector (Promega). A firefly luciferase expression vector driven by the first intron of Tnnc1 gene was constructed by replacing the CMV enhancer of pTriEx-1.1 vector (Novagen) with the first intron of Tnnc1 (~1.3 kb) followed by insertion of a restriction enzyme-digested PCR product of firefly luciferase gene from pGL3-basic vector into the appropriate restriction sites. A *Renilla *luciferase expresion vector driven by CMV promoter (pcDNA-Rluc) was produced by inserting the *Renilla *luciferase gene, which was obtained by digesting pRL-TK vector (Promega) with NheI and XbaI, into pcDNA3.1/Zeo(+) vector (Invitrogen).

Primers used were listed in Additional file 10, Table S5.

### Reporter assay

The reporter plasmids (see above) were co-transfected with the *Renilla *luciferase vector (pcDNA-Rluc) into mouse fetal primary myoblasts using Lipofectamine 2000 (Invitrogen) at 1:1.5 ratio of DNA and Lipofectamine. Twenty four hours after transfection, cells were washed with PBS once, and switched to DM. Cells were incubated further for 48 h, and firefly and *Renilla *luciferase activities were measured using Dual-Glo Luciferase Assay System (Promega) and a luminometer (LumiCount; Packard) according to the manufacturer's instructions. Statistical analyses were performed using the two-tailed Student's t-tests. As a negative control, pGL3-basic was used.

### Immunohistochemistry

Lower hindlimbs were collected from E18.5 embryos and one week old mice (P7), and TA and EDL muscles were collected from four month-old mice and embedded in Tissue Freezing Medium (Triangle Biomedical Sciences) for cryostat sectioning. Sections (10 μm) were fixed in 4% paraformaldehyde and processed for immunohistochemistry. As primary antibodies, rabbit polyclonal Sox6 antibody (ab30455, Abcam) at 400 fold dilution and mouse monoclonal MyHC-β antibody at 100 fold dilution (NOQ7.5.4D, Sigma-Aldrich) were used. As secondary antibodies, Alexa Fluor 555-conjugated goat anti-rabbit IgG (A-21429, Invitrogen) and Alexa Fluor 488-conjugated goat anti-mouse IgG (A-11059, Invitrogen) were used. DAPI staining was performed to visualize nucleus. Images were obtained at the confocal microscope facility at the UC Davis Genome and Biomedical Sciences Facility.

### Animal experiments

The animal studies were carried out under the guidance issued by the University of California, Davis.

## Authors' contributions

CIA performed ChIP-seq, ChIP-qPCR, RT-qPCR, plasmid construction, reporter assays, and contributed to writing the manuscript. YD performed immunostaining, RT-qPCR, and Western blotting. NH conceived, designed and supervised the study, and contributed to writing the manuscript. All authors read and approved the final manuscript.

## Supplementary Material

Additional file 1 **Figure S1 Relative mRNA levels of *Sox6, Prox1, Tead1, Tead4, Tcf4, Hdac9*, and *Hdac11 *in Sox6^f/f ^muscles. A**. *Sox6 *mRNA levels were determined in the adult EDL, TA, Gas, and Sol muscles using RT-qPCR and relative expression levels to the soleus in individual animals were calculated. Three 2 month-old and two three month-old Sox6^f/f ^mice were examined (*n *= 5). The error bars indicate standard error of the mean. The p-value for differential expression between the EDL and the soleus was 0.07. **B**. *Prox1 *mRNA levels were determined same as described for *Sox6 *(*n *= 3; two 2 month-old and one 3 month-old Sox6^f/f ^mice). **C-G**. For *Tead1, Tead4, Tcf4, Hdac9*, and *Hdac11*, data from EDL, TA, Gas were pooled and compared against soleus (*n *= 3). Click here for file

Additional file 2 **Table S1 Pol II binding data presented in Figure 4**. Full list of Pol II binding levels to the Sox6 peak-associated genes measured in RPKM are shown. Click here for file

Additional file 3 **Figure S2 Examples of Sox6 and Pol II binding events detected by ChIP-seq**. ChIP-seq tracks from two data sets of Sox6 (Sox6-1 and Sox6-2) are shown together with Pol II track (Pol II) of the 2.8 million read data (see the Methods section for details). Common Sox6 binding peaks between the two data sets are indicated as black bars (Sox6 peak). Chromosomal positions (mouse NCBI37/mm9 assembly) as well as sequence conservation (Vertebrate Cons) are presented above and below the ChIP-seq plots, respectively. **A**. *Myh1 *and *Myh2*, **B**. *Myh6 *and *Myh7*, **C**. *MyHC7b*, **D**. *Tnnc1*, **E**. *Tnni1*, **F**. *Prox1*, **G**. *Sox6*, **H**. *Tead1*, **I**. *Tead4*, **J**. *Nfatc3*, **K**. *Tcf4*, **L**. *Hdac9*, and **M**. *Hdac11*. Click here for file

Additional file 4 **Figure S3 Validation of Sox6 binding**. A total of 28 Sox6 peaks identified for the 19 genes discussed in the text were verified by ChIP-qPCR (ChIP followed by quantitative PCR). The peak profiles are summarized in Additional file 3, Figure S2A-M. ChIP was performed using wild type myotubes and Sox6 antibody as described in the Methods section, and enrichment was quantified by qPCR using the primers designed to amplify each Sox6 binding site (Additional file 6, Table S3). As a negative control, an intergenic region without a Sox6 peak was used. Fold enrichment over a negative control region (Intergenic) are shown. The intergenic region showed no enrichment. Data are represented as mean ± SD (*n *= 3). (*) *P *< 0.05; (**) *P *< .005. **A**. Enrichment of the Sox6 binding sites associated with sarcomeric protein genes. **B**. Enrichment of the Sox binding sites associated with transcription regulatory genes. Numbers in the parentheses below gene symbol indicate relative positions (5' to 3') of multiple Sox6 binding sites associated to the gene. For *Tcf4*, only intragenic binding sites (see Additional file 3, Figure S2K) were tested. Click here for file

Additional file 5 **Table S2 Biological processes enriched among genes associated with Sox6 peaks**. Full list of Gene Ontology (GO) biological process terms identified by DAVID are shown. Click here for file

Additional files 6 **Table S3 Primers used for ChIP-qPCR**. Click here for file

Additional files 7 **Table S4 TaqMan probes used for RT-qPCR**. Click here for file

Additional file 8 **Figure S4 Relative mRNA levels of the genes presented in Table **1. Relative mRNA levels against β-actin in TA, EDL, gastrocnemius (Gas), and soleus (Sol) of control (Sox6^f/f^) and Sox6 knockout (KO, Sox6^f/f; ^Myf5-Cre) mice were calculated using the formula 2^-ΔCt^. A two and three month-old mice (2 mo and 3 mo, respectively) were analyzed. **A**. Relative mRNA level of Sox6. **B**. Relative mRNA level of *Myh7*, *Myh4*, *Ppargc1a*, and *Sdha*. Click here for file

Additional file 9 **Figure S5 Relative mRNA levels of the genes presented in Table **2**and **4. Relative mRNA levels against β-actin in control (Sox6^f/f^) and Sox6 knockout (KO, Sox6^f/f; ^MCK-Cre) mice were calculated using the formula 2^-ΔCt^. Two 2 month-old mice (mouse ID# 1 and 2) and one 3 old-month mouse (mouse ID# 3) were analyzed. **A**. Relative mRNA level of Sox6 in TA, EDL, gastrocnemius (Gas), soleus (Sol), and the heart. **B**. Relative mRNA level of contractile protein genes in TA, EDL, Gas, and Sol. **C**. Relative mRNA level of transcriptional regulatory genes in TA, EDL, Gas, and Sol. **D**. Relative mRNA level of metabolism related genes and acetylcholine receptor genes in TA, EDL, Gas, and Sol. **E**. Relative mRNA level of *Myh6*, *Myh7*, *Acta1*, and *Ppargc1a *in the heart. *: not determined. #: undetected. Click here for file

Additional file 10 **Table S5 Primers used for plasmid construction**. Click here for file

## References

[B1] Zierath JR Hawley JA Skeletal muscle fiber type: influence on contractile and metabolic properties PLoS Biol 2004 2 e348 10.1371/journal.pbio.0020348 15486583 PMC521732

[B2] Pette D Staron RS Myosin isoforms, muscle fiber types, and transitions Microsc Res Tech 2000 50 500 9 10.1002/1097-0029(20000915)50:6<500::AID-JEMT7>3.0.CO;2-7 10998639

[B3] Schiaffino S Sandri M Murgia M Activity-dependent signaling pathways controlling muscle diversity and plasticity Physiology (Bethesda) 2007 22 269 78 10.1152/physiol.00009.2007 17699880

[B4] Gunning P Hardeman E Multiple mechanisms regulate muscle fiber diversity FASEB J 1991 5 3064 70 1835946 10.1096/fasebj.5.15.1835946

[B5] Lu BD Allen DL Leinwand LA Lyons GE Spatial and temporal changes in myosin heavy chain gene expression in skeletal muscle development Dev Biol 1999 216 312 26 10.1006/dbio.1999.9488 10588881

[B6] Agbulut O Noirez P Beaumont F Butler-Browne G Myosin heavy chain isoforms in postnatal muscle development of mice Biol Cell 2003 95 399 406 10.1016/S0248-4900(03)00087-X 14519557

[B7] Whalen RG Johnstone D Bryers PS Butler-Browne GS Ecob MS Jaros E A developmentally regulated disappearance of slow myosin in fast-type muscles of the mouse FEBS Lett 1984 177 51 6 10.1016/0014-5793(84)80979-5 6389176

[B8] Buller AJ Eccles JC Eccles RM Interactions between motoneurones and muscles in respect of the characteristic speeds of their responses J Physiol 1960 150 417 39 13805874 10.1113/jphysiol.1960.sp006395 PMC1363172

[B9] Goldspink G Selective gene expression during adaptation of muscle in response to different physiological demands Comp Biochem Physiol B Biochem Mol Biol 1998 120 5 15 10.1016/S0305-0491(98)00018-2 9787775

[B10] Lomo T Westgaard RH Dahl HA Contractile properties of muscle: control by pattern of muscle activity in the rat Proc R Soc Lond B Biol Sci 1974 187 99 103 10.1098/rspb.1974.0064 4153974

[B11] Pette D Vrbova G What does chronic electrical stimulation teach us about muscle plasticity? Muscle Nerve 1999 22 666 77 10.1002/(SICI)1097-4598(199906)22:6<666::AID-MUS3>3.0.CO;2-Z 10366220

[B12] Hagiwara N Ma B Ly A Slow and fast fiber isoform gene expression is systematically altered in skeletal muscle of the Sox6 mutant, p100H Dev Dyn 2005 234 301 11 10.1002/dvdy.20535 16124007

[B13] Hagiwara N Yeh M Liu A Sox6 is required for normal fiber type differentiation of fetal skeletal muscle in mice Dev Dyn 2007 236 2062 76 10.1002/dvdy.21223 17584907

[B14] Bowles J Schepers G Koopman P Phylogeny of the SOX family of developmental transcription factors based on sequence and structural indicators Dev Biol 2000 227 239 55 10.1006/dbio.2000.9883 11071752

[B15] Hagiwara N Sox6, Jack of all trades: A versatile regulatory protein in vertebrate development Dev Dyn 2011 240 1311 21 10.1002/dvdy.22639 21495113 PMC3092843

[B16] Kamachi Y Uchikawa M Kondoh H Pairing SOX off: with partners in the regulation of embryonic development Trends Genet 2000 16 182 7 10.1016/S0168-9525(99)01955-1 10729834

[B17] Wegner M From head to toes: the multiple facets of Sox proteins Nucleic Acids Res 1999 27 1409 20 10.1093/nar/27.6.1409 10037800 PMC148332

[B18] Cohen-Barak O Hagiwara N Arlt MF Horton JP Brilliant MH Cloning, characterization and chromosome mapping of the human SOX6 gene Gene 2001 265 157 64 10.1016/S0378-1119(01)00346-8 11255018

[B19] Wilson M Koopman P Matching SOX: partner proteins and co-factors of the SOX family of transcriptional regulators Curr Opin Genet Dev 2002 12 441 6 10.1016/S0959-437X(02)00323-4 12100890

[B20] Han Y Lefebvre V L-Sox5 and Sox6 drive expression of the aggrecan gene in cartilage by securing binding of Sox9 to a far-upstream enhancer Mol Cell Biol 2008 28 4999 5013 10.1128/MCB.00695-08 18559420 PMC2519711

[B21] Lefebvre V Li P de Crombrugghe B A new long form of Sox5 (L-Sox5), Sox6 and Sox9 are coexpressed in chondrogenesis and cooperatively activate the type II collagen gene EMBO J 1998 17 5718 33 10.1093/emboj/17.19.5718 9755172 PMC1170900

[B22] Nagy A Kenesi E Rentsendorj O Molnar A Szenasi T Sinko I Zvara A Oommen ST Barta E Puskas LG Evolutionarily conserved, growth plate zone-specific regulation of the matrilin-1 promoter: L-Sox5/Sox6 and Nfi factors bound near TATA finely tune activation by Sox9 Mol Cell Biol 2011 31 686 99 10.1128/MCB.00019-10 21173167 PMC3028657

[B23] Iguchi H Urashima Y Inagaki Y Ikeda Y Okamura M Tanaka T Uchida A Yamamoto TT Kodama T Sakai J SOX6 suppresses cyclin D1 promoter activity by interacting with betacatenin and histone deacetylase 1, and its down-regulation induces pancreatic beta-cell proliferation J Biol Chem 2007 282 19052 61 10.1074/jbc.M700460200 17412698

[B24] Murakami A Ishida S Thurlow J Revest JM Dickson C SOX6 binds CtBP2 to repress transcription from the Fgf-3 promoter Nucleic Acids Res 2001 29 3347 55 10.1093/nar/29.16.3347 11504872 PMC55854

[B25] Dumitriu B Dy P Smits P Lefebvre V Generation of mice harboring a Sox6 conditional null allele Genesis 2006 44 219 24 10.1002/dvg.20210 16652367

[B26] Hagiwara N Klewer SE Samson RA Erickson DT Lyon MF Brilliant MH Sox6 is a candidate gene for p100H myopathy, heart block, and sudden neonatal death Proc Natl Acad Sci USA 2000 97 4180 5 10.1073/pnas.97.8.4180 10760285 PMC18189

[B27] Smits P Li P Mandel J Zhang Z Deng JM Behringer RR de Crombrugghe B Lefebvre V The transcription factors L-Sox5 and Sox6 are essential for cartilage formation Dev Cell 2001 1 277 90 10.1016/S1534-5807(01)00003-X 11702786

[B28] Tallquist MD Weismann KE Hellstrom M Soriano P Early myotome specification regulates PDGFA expression and axial skeleton development Development 2000 127 5059 70 11060232 10.1242/dev.127.23.5059

[B29] Richard AF Demignon J Sakakibara I Pujol J Favier M Strochlic L Le Grand F Sgarioto N Guernec A Schmitt A Genesis of muscle fiber-type diversity during mouse embryogenesis relies on Six1 and Six4 gene expression Dev Biol 2011 359 303 20 10.1016/j.ydbio.2011.08.010 21884692

[B30] Giguere V Transcriptional control of energy homeostasis by the estrogen-related receptors Endocr Rev 2008 29 677 96 10.1210/er.2008-0017 18664618

[B31] Lin JD Handschin C Spiegelman BM Metabolic control through the PGC-1 family of transcription coactivators Cell Metabolism 2005 1 361 370 10.1016/j.cmet.2005.05.004 16054085

[B32] Berchtold MW Brinkmeier H Muntener M Calcium ion in skeletal muscle: its crucial role for muscle function, plasticity, and disease Physiol Rev 2000 80 1215 65 10893434 10.1152/physrev.2000.80.3.1215

[B33] Lin J Wu H Tarr PT Zhang CY Wu Z Boss O Michael LF Puigserver P Isotani E Olson EN Transcriptional co-activator PGC-1 alpha drives the formation of slow-twitch muscle fibres Nature 2002 418 797 801 10.1038/nature00904 12181572

[B34] Quiat D Voelker KA Pei J Grishin NV Grange RW Bassel-Duby R Olson EN Concerted regulation of myofiber-specific gene expression and muscle performance by the transcriptional repressor Sox6 Proc Natl Acad Sci USA 2011 108 10196 201 10.1073/pnas.1107413108 21633012 PMC3121857

[B35] Bailey TL Elkan C Fitting a mixture model by expectation maximization to discover motifs in biopolymers Proc Int Conf Intell Syst Mol Biol 1994 2 28 36 7584402

[B36] Cao Y Yao Z Sarkar D Lawrence M Sanchez GJ Parker MH MacQuarrie KL Davison J Morgan MT Ruzzo WL Genome-wide MyoD binding in skeletal muscle cells: a potential for broad cellular reprogramming Dev Cell 2010 18 662 74 10.1016/j.devcel.2010.02.014 20412780 PMC2910615

[B37] Philipot O Joliot V Ait-Mohamed O Pellentz C Robin P Fritsch L Ait-Si-Ali S The core binding factor CBF negatively regulates skeletal muscle terminal differentiation PLoS One 2010 5 e9425 10.1371/journal.pone.0009425 20195544 PMC2828485

[B38] Yoshida T MCAT elements and the TEF-1 family of transcription factors in muscle development and disease Arterioscler Thromb Vasc Biol 2008 28 8 17 17962623 10.1161/ATVBAHA.107.155788

[B39] Brookes E Pombo A Modifications of RNA polymerase II are pivotal in regulating gene expression states EMBO Rep 2009 10 1213 9 10.1038/embor.2009.221 19834511 PMC2775184

[B40] Mortazavi A Williams BA McCue K Schaeffer L Wold B Mapping and quantifying mammalian transcriptomes by RNA-Seq Nat Methods 2008 5 621 8 10.1038/nmeth.1226 18516045 PMC13303166

[B41] Parmacek MS Ip HS Jung F Shen T Martin JF Vora AJ Olson EN Leiden JM A novel myogenic regulatory circuit controls slow/cardiac troponin C gene transcription in skeletal muscle Mol Cell Biol 1994 14 1870 85 8114720 10.1128/mcb.14.3.1870-1885.1994 PMC358545

[B42] Armand AS Bourajjaj M Martinez-Martinez S el Azzouzi H da Costa Martins PA Hatzis P Seidler T Redondo JM De Windt LJ Cooperative synergy between NFAT and MyoD regulates myogenin expression and myogenesis J Biol Chem 2008 283 29004 10 10.1074/jbc.M801297200 18676376 PMC2662004

[B43] Delling U Tureckova J Lim HW De Windt LJ Rotwein P Molkentin JD A calcineurin-NFATc3-dependent pathway regulates skeletal muscle differentiation and slow myosin heavy-chain expression Mol Cell Biol 2000 20 6600 11 10.1128/MCB.20.17.6600-6611.2000 10938134 PMC86143

[B44] Haberland M Arnold MA McAnally J Phan D Kim Y Olson EN Regulation of HDAC9 gene expression by MEF2 establishes a negative-feedback loop in the transcriptional circuitry of muscle differentiation Mol Cell Biol 2007 27 518 25 10.1128/MCB.01415-06 17101791 PMC1800816

[B45] Kegley KM Gephart J Warren GL Pavlath GK Altered primary myogenesis in NFATC3(-/-) mice leads to decreased muscle size in the adult Dev Biol 2001 232 115 26 10.1006/dbio.2001.0179 11254352

[B46] Risebro CA Searles RG Melville AA Ehler E Jina N Shah S Pallas J Hubank M Dillard M Harvey NL Prox1 maintains muscle structure and growth in the developing heart Development 2009 136 495 505 10.1242/dev.030007 19091769 PMC2655234

[B47] Roy S Wolff C Ingham PW The u-boot mutation identifies a Hedgehog-regulated myogenic switch for fiber-type diversification in the zebrafish embryo Genes Dev 2001 15 1563 76 10.1101/gad.195801 11410536 PMC312718

[B48] Singh R Bhasin S Braga M Artaza JN Pervin S Taylor WE Krishnan V Sinha SK Rajavashisth TB Jasuja R Regulation of myogenic differentiation by androgens: cross talk between androgen receptor/beta-catenin and follistatin/transforming growth factor-beta signaling pathways Endocrinology 2009 150 1259 68 18948405 10.1210/en.2008-0858 PMC2654730

[B49] van der Velden JL Schols AM Willems J Kelders MC Langen RC Glycogen synthase kinase 3 suppresses myogenic differentiation through negative regulation of NFATc3 J Biol Chem 2008 283 358 66 17977834 10.1074/jbc.M707812200

[B50] Gregoretti IV Lee YM Goodson HV Molecular evolution of the histone deacetylase family: functional implications of phylogenetic analysis J Mol Biol 2004 338 17 31 10.1016/j.jmb.2004.02.006 15050820

[B51] Gao L Cueto MA Asselbergs F Atadja P Cloning and functional characterization of HDAC11, a novel member of the human histone deacetylase family J Biol Chem 2002 277 25748 55 10.1074/jbc.M111871200 11948178

[B52] Cantu C Grande V Alborelli I Cassinelli L Cantu I Colzani MT Ierardi R Ronzoni L Cappellini MD Ferrari G A highly conserved SOX6 double binding site mediates SOX6 gene downregulation in erythroid cells Nucleic Acids Res 2011 39 486 501 10.1093/nar/gkq819 20852263 PMC3025548

[B53] Andrechek ER Hardy WR Girgis-Gabardo AA Perry RL Butler R Graham FL Kahn RC Rudnicki MA Muller WJ ErbB2 is required for muscle spindle and myoblast cell survival Mol Cell Biol 2002 22 4714 22 10.1128/MCB.22.13.4714-4722.2002 12052879 PMC133917

[B54] Wang J Wilhelmsson H Graff C Li H Oldfors A Rustin P Bruning JC Kahn CR Clayton DA Barsh GS Dilated cardiomyopathy and atrioventricular conduction blocks induced by heart-specific inactivation of mitochondrial DNA gene expression Nat Genet 1999 21 133 7 10.1038/5089 9916807

[B55] Schiaffino S Fibre types in skeletal muscle: a personal account Acta Physiol (Oxf) 2010 199 451 63 10.1111/j.1748-1716.2010.02130.x 20353491

[B56] Calderon JC Bolanos P Caputo C Myosin heavy chain isoform composition and Ca(2+) transients in fibres from enzymatically dissociated murine soleus and extensor digitorum longus muscles J Physiol 2010 588 267 79 10.1113/jphysiol.2009.180893 19884322 PMC2821564

[B57] LaFramboise WA Daood MJ Guthrie RD Moretti P Schiaffino S Ontell M Electrophoretic separation and immunological identification of type 2X myosin heavy chain in rat skeletal muscle Biochim Biophys Acta 1990 1035 109 12 10.1016/0304-4165(90)90181-U 2383576

[B58] Missias AC Chu GC Klocke BJ Sanes JR Merlie JP Maturation of the acetylcholine receptor in skeletal muscle: regulation of the AChR gamma-to-epsilon switch Dev Biol 1996 179 223 38 10.1006/dbio.1996.0253 8873766

[B59] Hughes SM Koishi K Rudnicki M Maggs AM MyoD protein is differentially accumulated in fast and slow skeletal muscle fibres and required for normal fibre type balance in rodents Mech Dev 1997 61 151 63 10.1016/S0925-4773(96)00631-4 9076685

[B60] Hughes SM Taylor JM Tapscott SJ Gurley CM Carter WJ Peterson CA Selective accumulation of MyoD and myogenin mRNAs in fast and slow adult skeletal muscle is controlled by innervation and hormones Development 1993 118 1137 47 8269844 10.1242/dev.118.4.1137

[B61] Lyons GE Schiaffino S Sassoon D Barton P Buckingham M Developmental regulation of myosin gene expression in mouse cardiac muscle J Cell Biol 1990 111 2427 36 10.1083/jcb.111.6.2427 2277065 PMC2116419

[B62] Ruzicka DL Schwartz RJ Sequential activation of alpha-actin genes during avian cardiogenesis: vascular smooth muscle alpha-actin gene transcripts mark the onset of cardiomyocyte differentiation J Cell Biol 1988 107 2575 86 10.1083/jcb.107.6.2575 3204121 PMC2115638

[B63] Lehman JJ Boudina S Banke NH Sambandam N Han X Young DM Leone TC Gross RW Lewandowski ED Abel ED The transcriptional coactivator PGC-1alpha is essential for maximal and efficient cardiac mitochondrial fatty acid oxidation and lipid homeostasis Am J Physiol Heart Circ Physiol 2008 295 H185 96 10.1152/ajpheart.00081.2008 18487436 PMC2494758

[B64] Rowe GC Jiang A Arany Z PGC-1 coactivators in cardiac development and disease Circ Res 2010 107 825 38 10.1161/CIRCRESAHA.110.223818 20884884 PMC2955978

[B65] Russell LK Mansfield CM Lehman JJ Kovacs A Courtois M Saffitz JE Medeiros DM Valencik ML McDonald JA Kelly DP Cardiac-specific induction of the transcriptional coactivator peroxisome proliferator-activated receptor gamma coactivator-1alpha promotes mitochondrial biogenesis and reversible cardiomyopathy in a developmental stage-dependent manner Circ Res 2004 94 525 33 10.1161/01.RES.0000117088.36577.EB 14726475

[B66] Schulz RA Yutzey KE Calcineurin signaling and NFAT activation in cardiovascular and skeletal muscle development Dev Biol 2004 266 1 16 10.1016/j.ydbio.2003.10.008 14729474

[B67] Nakayama M Stauffer J Cheng J Banerjee-Basu S Wawrousek E Buonanno A Common core sequences are found in skeletal muscle slow- and fast-fiber-type-specific regulatory elements Mol Cell Biol 1996 16 2408 17 8628309 10.1128/mcb.16.5.2408 PMC231230

[B68] Bell ML Buvoli M Leinwand LA Uncoupling of expression of an intronic microRNA and its myosin host gene by exon skipping Mol Cell Biol 2010 30 1937 45 10.1128/MCB.01370-09 20154144 PMC2849460

[B69] Blow MJ McCulley DJ Li Z Zhang T Akiyama JA Holt A Plajzer-Frick I Shoukry M Wright C Chen F ChIP-Seq identification of weakly conserved heart enhancers Nat Genet 2010 42 806 10 10.1038/ng.650 20729851 PMC3138496

[B70] Giger JM Haddad F Qin AX Baldwin KM In vivo regulation of the beta-myosin heavy chain gene in soleus muscle of suspended and weight-bearing rats Am J Physiol Cell Physiol 2000 278 C1153 61 10837343 10.1152/ajpcell.2000.278.6.C1153

[B71] Rudnicki MA Schnegelsberg PN Stead RH Braun T Arnold HH Jaenisch R MyoD or Myf-5 is required for the formation of skeletal muscle Cell 1993 75 1351 9 10.1016/0092-8674(93)90621-V 8269513

[B72] Weintraub H Davis R Tapscott S Thayer M Krause M Benezra R Blackwell TK Turner D Rupp R Hollenberg S The myoD gene family: nodal point during specification of the muscle cell lineage Science 1991 251 761 6 10.1126/science.1846704 1846704

[B73] Shia WJ Pattenden SG Workman JL Histone H4 lysine 16 acetylation breaks the genome's silence Genome Biol 2006 7 217 10.1186/gb-2006-7-5-217 16689998 PMC1779524

[B74] McKinsey TA Zhang CL Olson EN Control of muscle development by dueling HATs and HDACs Curr Opin Genet Dev 2001 11 497 504 10.1016/S0959-437X(00)00224-0 11532390

[B75] Larkin SB Farrance IK Ordahl CP Flanking sequences modulate the cell specificity of MCAT elements Mol Cell Biol 1996 16 3742 55 8668191 10.1128/mcb.16.7.3742 PMC231370

[B76] Wang X Blagden C Fan J Nowak SJ Taniuchi I Littman DR Burden SJ Runx1 prevents wasting, myofibrillar disorganization, and autophagy of skeletal muscle Genes Dev 2005 19 1715 22 10.1101/gad.1318305 16024660 PMC1176009

[B77] Zhu X Yeadon JE Burden SJ AML1 is expressed in skeletal muscle and is regulated by innervation Mol Cell Biol 1994 14 8051 7 7969143 10.1128/mcb.14.12.8051-8057.1994 PMC359343

[B78] Wheeler JC Shigesada K Gergen JP Ito Y Mechanisms of transcriptional regulation by Runt domain proteins Semin Cell Dev Biol 2000 11 369 75 10.1006/scdb.2000.0184 11105901

[B79] Trimborn T Gribnau J Grosveld F Fraser P Mechanisms of developmental control of transcription in the murine alpha- and beta-globin loci Genes Dev 1999 13 112 24 10.1101/gad.13.1.112 9887104 PMC316369

[B80] Yi Z Cohen-Barak O Hagiwara N Kingsley PD Fuchs DA Erickson DT Epner EM Palis J Brilliant MH Sox6 directly silences epsilon globin expression in definitive erythropoiesis PLoS Genet 2006 2 e14 10.1371/journal.pgen.0020014 16462943 PMC1359074

[B81] Xu J Sankaran VG Ni M Menne TF Puram RV Kim W Orkin SH Transcriptional silencing of {gamma}-globin by BCL11A involves long-range interactions and cooperation with SOX6 Genes Dev 2010 24 783 98 10.1101/gad.1897310 20395365 PMC2854393

[B82] Sutherland H Bickmore WA Transcription factories: gene expression in unions? Nat Rev Genet 2009 10 457 66 19506577 10.1038/nrg2592

[B83] Gan Q Yoshida T Li J Owens GK Smooth muscle cells and myofibroblasts use distinct transcriptional mechanisms for smooth muscle alpha-actin expression Circ Res 2007 101 883 92 10.1161/CIRCRESAHA.107.154831 17823374

[B84] Stewart AF Richard CW Suzow J Stephan D Weremowicz S Morton CC Adra CN Cloning of human RTEF-1, a transcriptional enhancer factor-1-related gene preferentially expressed in skeletal muscle: evidence for an ancient multigene family Genomics 1996 37 68 76 10.1006/geno.1996.0522 8921372

[B85] Stewart AF Suzow J Kubota T Ueyama T Chen HH Transcription factor RTEF-1 mediates alpha1-adrenergic reactivation of the fetal gene program in cardiac myocytes Circ Res 1998 83 43 9 9670917 10.1161/01.res.83.1.43

[B86] Parra M Verdin E Regulatory signal transduction pathways for class IIa histone deacetylases Curr Opin Pharmacol 2010 10 454 60 10.1016/j.coph.2010.04.004 20447866

[B87] Mejat A Ramond F Bassel-Duby R Khochbin S Olson EN Schaeffer L Histone deacetylase 9 couples neuronal activity to muscle chromatin acetylation and gene expression Nat Neurosci 2005 8 313 21 10.1038/nn1408 15711539

[B88] von Hofsten J Elworthy S Gilchrist MJ Smith JC Wardle FC Ingham PW Prdm1- and Sox6- mediated transcriptional repression specifies muscle fibre type in the zebrafish embryo EMBO Rep 2008 9 683 9 10.1038/embor.2008.73 18535625 PMC2424280

[B89] Bakay M Zhao P Chen J Hoffman EP A web-accessible complete transcriptome of normal human and DMD muscle Neuromuscul Disord 2002 12 Suppl 1 S125 41 12206807 10.1016/s0960-8966(02)00093-7

[B90] Pedrosa-Domellof F Eriksson PO Butler-Browne GS Thornell LE Expression of alpha cardiac myosin heavy chain in mammalian skeletal muscle Experientia 1992 48 491 4 10.1007/BF01928171 1601115

[B91] Sciote JJ Horton MJ Rowlerson AM Link J Specialized cranial muscles: how different are they from limb and abdominal muscles? Cells Tissues Organs 2003 174 73 86 10.1159/000070576 12784043 PMC3848039

[B92] Niro C Demignon J Vincent S Liu Y Giordani J Sgarioto N Favier M Guillet-Deniau I Blais A Maire P Six1 and Six4 gene expression is necessary to activate the fast-type muscle gene program in the mouse primary myotome Dev Biol 2010 338 168 82 10.1016/j.ydbio.2009.11.031 19962975

[B93] Bartel DP MicroRNAs: target recognition and regulatory functions Cell 2009 136 215 33 10.1016/j.cell.2009.01.002 19167326 PMC3794896

[B94] Guo H Ingolia NT Weissman JS Bartel DP Mammalian microRNAs predominantly act to decrease target mRNA levels Nature 2010 466 835 40 10.1038/nature09267 20703300 PMC2990499

[B95] van Rooij E Liu N Olson EN MicroRNAs flex their muscles Trends Genet 2008 24 159 66 10.1016/j.tig.2008.01.007 18325627

[B96] van Rooij E Sutherland LB Qi X Richardson JA Hill J Olson EN Control of stressdependent cardiac growth and gene expression by a microRNA Science 2007 316 575 9 10.1126/science.1139089 17379774

[B97] McCarthy JJ Esser KA Peterson CA Dupont-Versteegden EE Evidence of MyomiR network regulation of beta-myosin heavy chain gene expression during skeletal muscle atrophy Physiol Genomics 2009 39 219 26 10.1152/physiolgenomics.00042.2009 19690046 PMC2789671

[B98] van Rooij E Quiat D Johnson BA Sutherland LB Qi X Richardson JA Kelm RJ Jr Olson EN A family of microRNAs encoded by myosin genes governs myosin expression and muscle performance Dev Cell 2009 17 662 73 10.1016/j.devcel.2009.10.013 19922871 PMC2796371

[B99] Rossi AC Mammucari C Argentini C Reggiani C Schiaffino S Two novel/ancient myosins in mammalian skeletal muscles: MYH14/7b and MYH15 are expressed in extraocular muscles and muscle spindles J Physiol 2010 588 353 64 10.1113/jphysiol.2009.181008 19948655 PMC2821527

[B100] Wang X Ono Y Tan SC Chai RJ Parkin C Ingham PW Prdm1a and miR-499 act sequentially to restrict Sox6 activity to the fast-twitch muscle lineage in the zebrafish embryo Development 2011 138 4399 404 10.1242/dev.070516 21880783

[B101] Biressi S Molinaro M Cossu G Cellular heterogeneity during vertebrate skeletal muscle development Dev Biol 2007 308 281 93 10.1016/j.ydbio.2007.06.006 17612520

[B102] Miike T Ohtani Y Tamari H Ishitsu T Une Y Muscle fiber type transformation in nemaline myopathy and congenital fiber type disproportion Brain Dev 1986 8 526 32 3799921 10.1016/s0387-7604(86)80098-5

[B103] Stupka N Plant DR Schertzer JD Emerson TM Bassel-Duby R Olson EN Lynch GS Activated calcineurin ameliorates contraction-induced injury to skeletal muscles of mdx dystrophic mice J Physiol 2006 575 645 56 10.1113/jphysiol.2006.108472 16793906 PMC1819459

[B104] Volpe P Damiani E Margreth A Pellegrini G Scarlato G Fast to slow change of myosin in nemaline myopathy: electrophoretic and immunologic evidence Neurology 1982 32 37 41 7198731 10.1212/wnl.32.1.37

[B105] Webster C Silberstein L Hays AP Blau HM Fast muscle fibers are preferentially affected in Duchenne muscular dystrophy Cell 1988 52 503 13 10.1016/0092-8674(88)90463-1 3342447

[B106] Rajabi M Kassiotis C Razeghi P Taegtmeyer H Return to the fetal gene program protects the stressed heart: a strong hypothesis Heart Fail Rev 2007 12 331 43 10.1007/s10741-007-9034-1 17516164

[B107] Nowak DE Tian B Brasier AR Two-step cross-linking method for identification of NFkappaB gene network by chromatin immunoprecipitation Biotechniques 2005 39 715 25 10.2144/000112014 16315372

[B108] Schmidt D Wilson MD Spyrou C Brown GD Hadfield J Odom DT ChIP-seq: using highthroughput sequencing to discover protein-DNA interactions Methods 2009 48 240 8 10.1016/j.ymeth.2009.03.001 19275939 PMC4052679

[B109] Langmead B Trapnell C Pop M Salzberg SL Ultrafast and memory-efficient alignment of short DNA sequences to the human genome Genome Biol 2009 10 R25 10.1186/gb-2009-10-3-r25 19261174 PMC2690996

[B110] Jothi R Cuddapah S Barski A Cui K Zhao K Genome-wide identification of in vivo protein-DNA binding sites from ChIP-Seq data Nucleic Acids Res 2008 36 5221 31 10.1093/nar/gkn488 18684996 PMC2532738

[B111] Blahnik KR Dou L O'Geen H McPhillips T Xu X Cao AR Iyengar S Nicolet CM Ludascher B Korf I Sole-Search: an integrated analysis program for peak detection and functional annotation using ChIP-seq data Nucleic Acids Res 2010 38 e13 10.1093/nar/gkp1012 19906703 PMC2817454

[B112] Salmon-Divon M Dvinge H Tammoja K Bertone P PeakAnalyzer: genome-wide annotation of chromatin binding and modification loci BMC Bioinformatics 2010 11 415 10.1186/1471-2105-11-415 20691053 PMC2923140

[B113] Kent WJ Sugnet CW Furey TS Roskin KM Pringle TH Zahler AM Haussler D The human genome browser at UCSC Genome Res 2002 12 996 1006 12045153 10.1101/gr.229102 PMC186604

[B114] Gupta S Stamatoyannopoulos JA Bailey TL Noble WS Quantifying similarity between motifs Genome Biol 2007 8 R24 10.1186/gb-2007-8-2-r24 17324271 PMC1852410

[B115] Dennis G Jr Sherman BT Hosack DA Yang J Gao W Lane HC Lempicki RA DAVID: Database for Annotation, Visualization, and Integrated Discovery Genome Biol 2003 4 P3 10.1186/gb-2003-4-5-p3 12734009 PMC193660

[B116] Huang DW Sherman BT Lempicki RA Systematic and integrative analysis of large gene lists using DAVID bioinformatics resources Nature Protocols 2009 4 44 57 19131956 10.1038/nprot.2008.211

[B117] Huey KA Haddad F Qin AX Baldwin KM Transcriptional regulation of the type I myosin heavy chain gene in denervated rat soleus Am J Physiol Cell Physiol 2003 284 C738 48 12444021 10.1152/ajpcell.00389.2002

[B118] Huey KA Roy RR Haddad F Edgerton VR Baldwin KM Transcriptional regulation of the type I myosin heavy chain promoter in inactive rat soleus Am J Physiol Cell Physiol 2002 282 C528 37 11832338 10.1152/ajpcell.00355.2001

[B119] Imai Y Matsushima Y Sugimura T Terada M A simple and rapid method for generating a deletion by PCR Nucleic Acids Res 1991 19 2785 10.1093/nar/19.10.2785 1645866 PMC328208

