# Supplementary material for: Genome-wide mapping of Sox6 binding sites in skeletal muscle reveals both direct and indirect regulation of muscle terminal differentiation by Sox6
Source: BMC Dev Biol. 2011 Oct 10;11:59. doi: 10.1186/1471-213X-11-59 (PMC3239296; doi:10.1186/1471-213X-11-59)
Supplement: Additional file 1 — Figure S1 Relative mRNA levels of Sox6, Prox1, Tead1, Tead4, Tcf4, Hdac9, and Hdac11 in Sox6f/f muscles. A. Sox6 mRNA levels were determined in the adult EDL, TA, Gas, and Sol muscles using RT-qPCR and relative expression levels to the soleus in individual animals were calculated. Three 2 month-old and two three month-old Sox6f/f mice were examined (n = 5). The error bars indicate standard error of the mean. The p-value for differential expression between the EDL and the soleus was 0.07. B. Prox1 mRNA levels were determined same as described for Sox6 (n = 3; two 2 month-old and one 3 month-old Sox6f/f mice). C-G. For Tead1, Tead4, Tcf4, Hdac9, and Hdac11, data from EDL, TA, Gas were pooled and compared against soleus (n = 3). [file 1471-213X-11-59-S1.PDF]

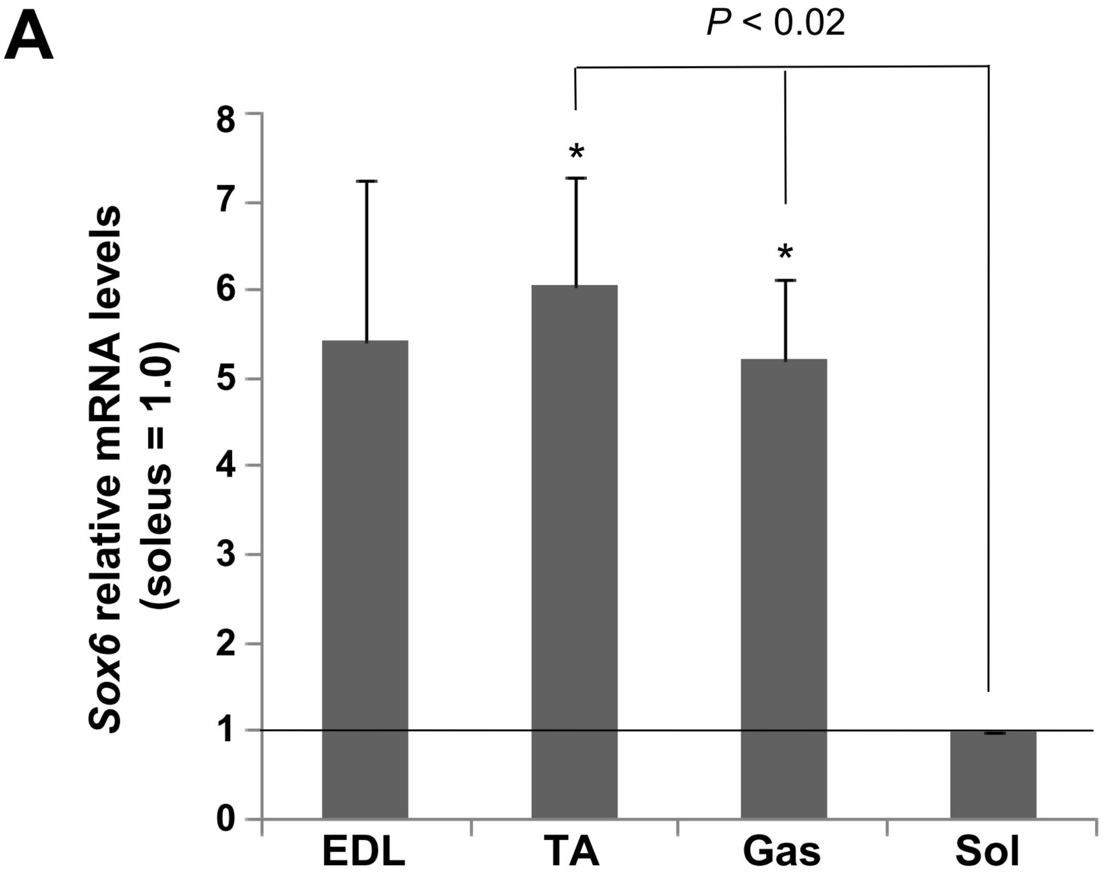
A
P < 0.02
Sox6 relative mRNA levels (soleus = 1.0)
8
7
6
5
4
3
2
1
0
*
*
EDL
TA
Gas
Sol

B

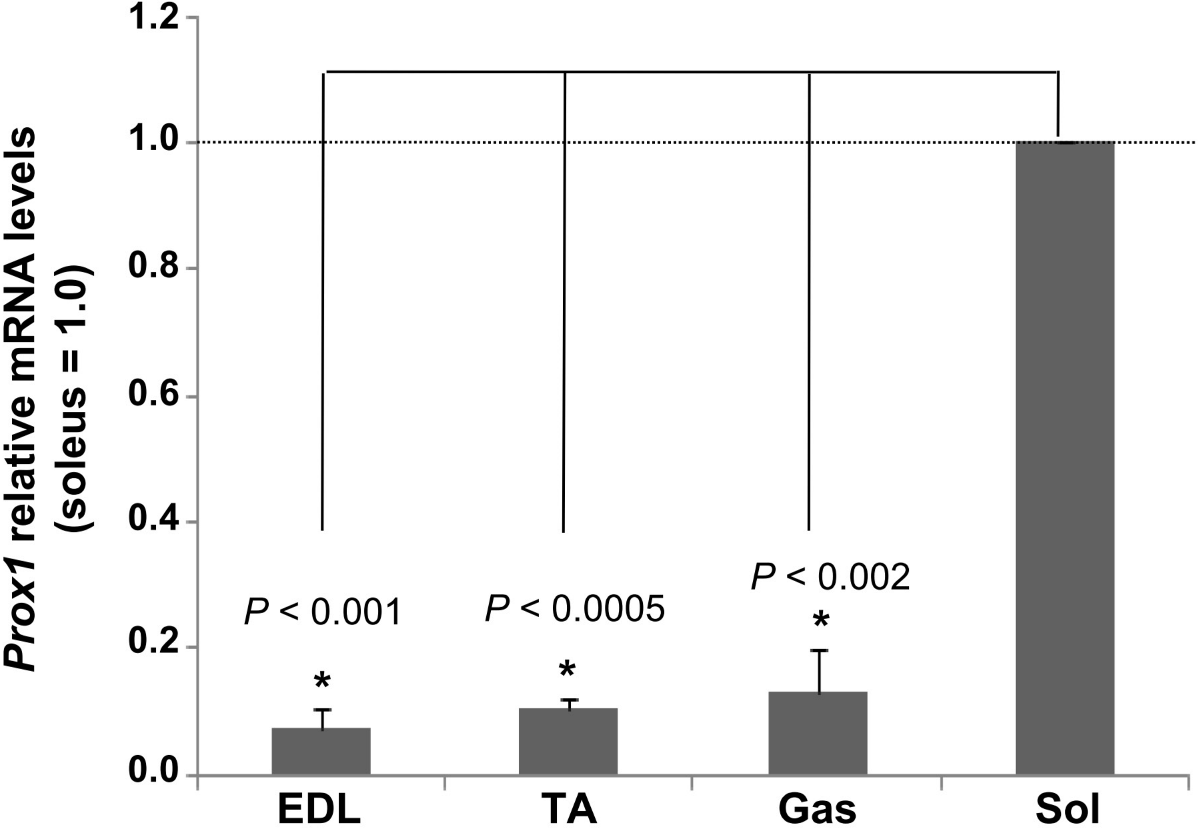
Prox1 relative mRNA levels (soleus = 1.0)
1.2
1.0
0.8
0.6
0.4
0.2
0.0
P < 0.001
P < 0.0005
P < 0.002
*
*
*
EDL
TA
Gas
Sol

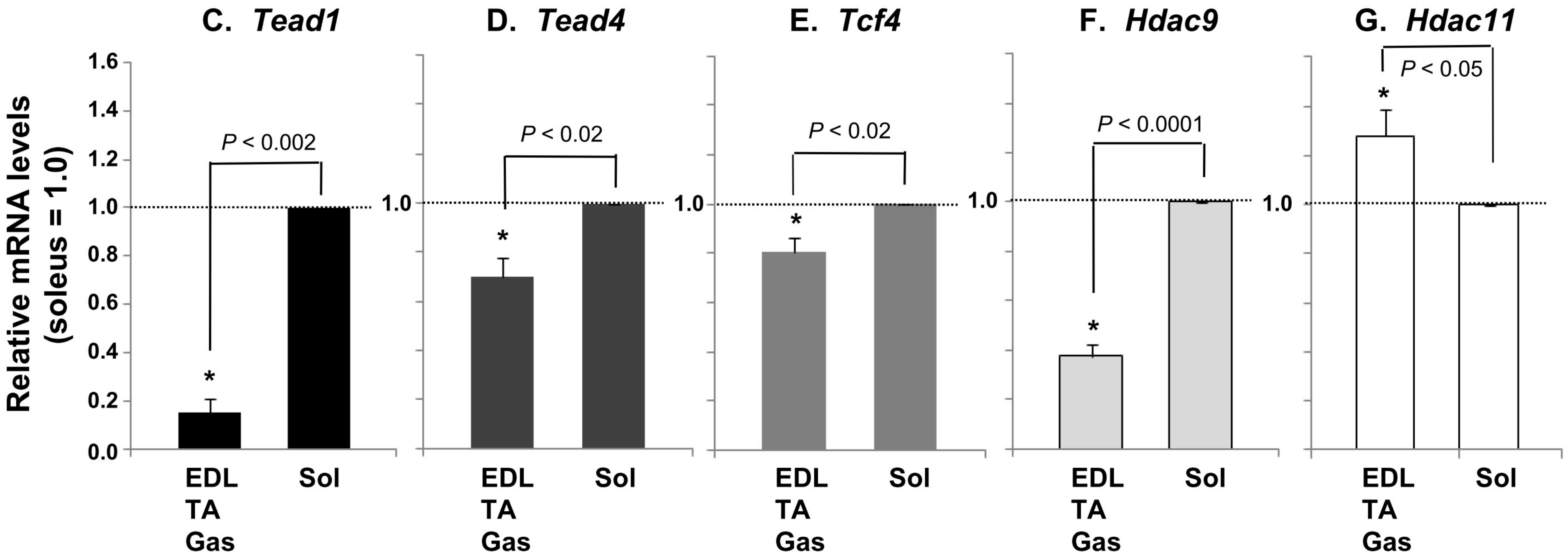

C. Tead1
D. Tead4
E. Tcf4
F. Hdac9
G. Hdac11
Relative mRNA levels (soleus = 1.0)
1.6
1.4
1.2
1.0
0.8
0.6
0.4
0.2
0.0
P < 0.002
P < 0.02
P < 0.02
P < 0.0001
P < 0.05
*
1.0
EDL TA Gas
Sol
