# Supplementary figures and images for: Genome-wide mapping of Sox6 binding sites in skeletal muscle reveals both direct and indirect regulation of muscle terminal differentiation by Sox6
Source: BMC Dev Biol. 2011 Oct 10;11:59. doi: 10.1186/1471-213X-11-59 (PMC3239296; doi:10.1186/1471-213X-11-59)

**A**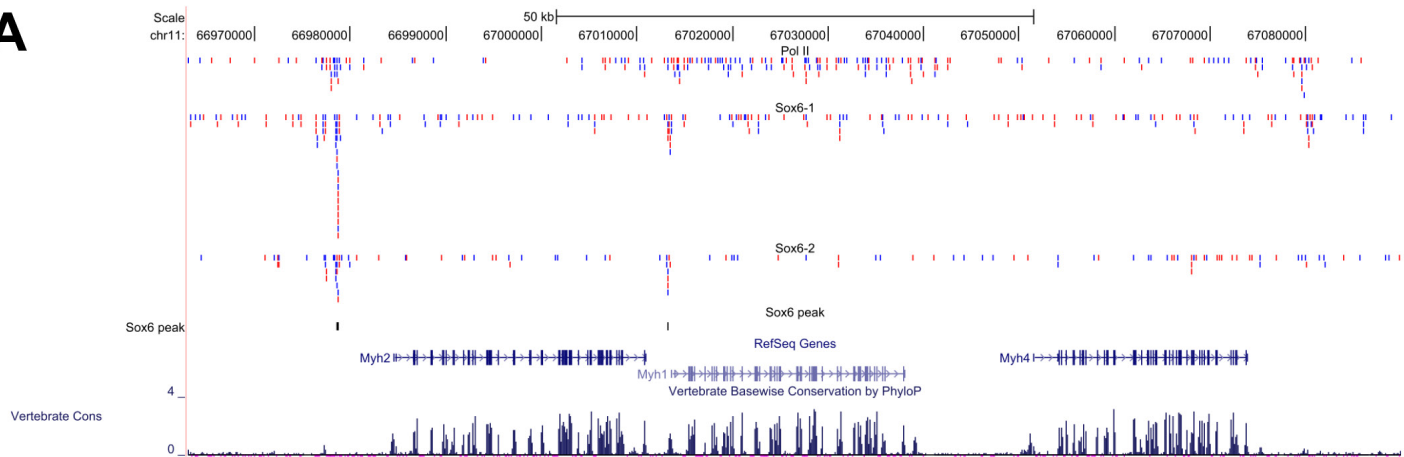**B**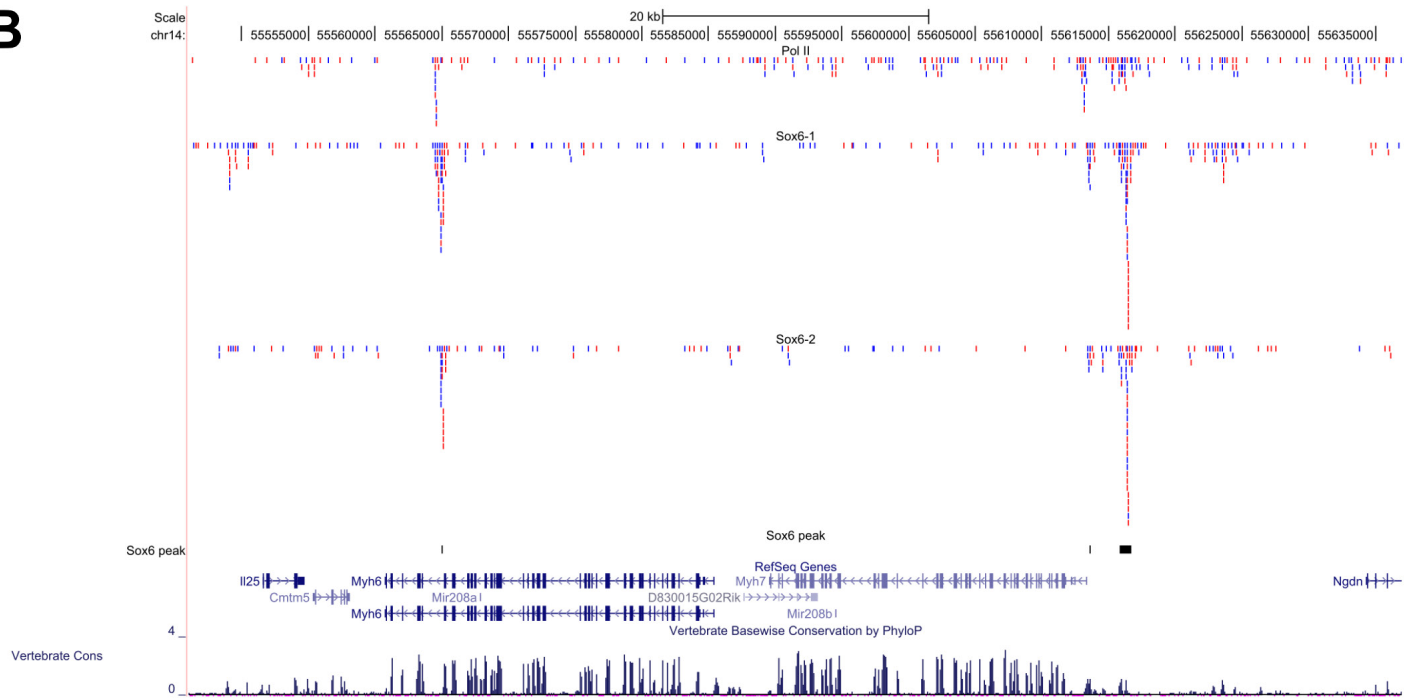

**C**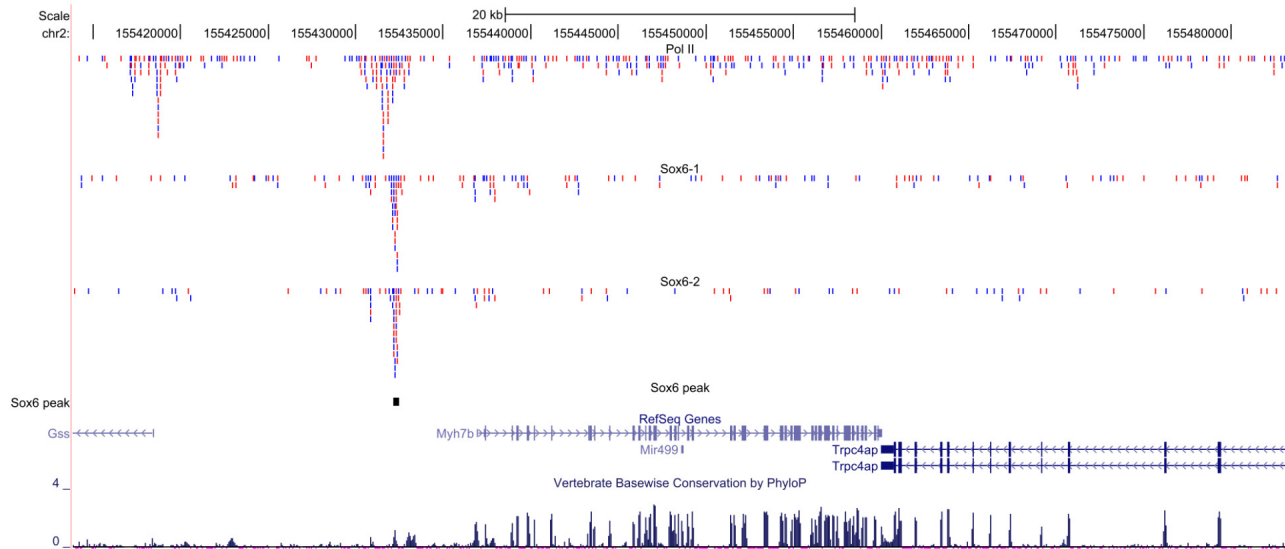**D**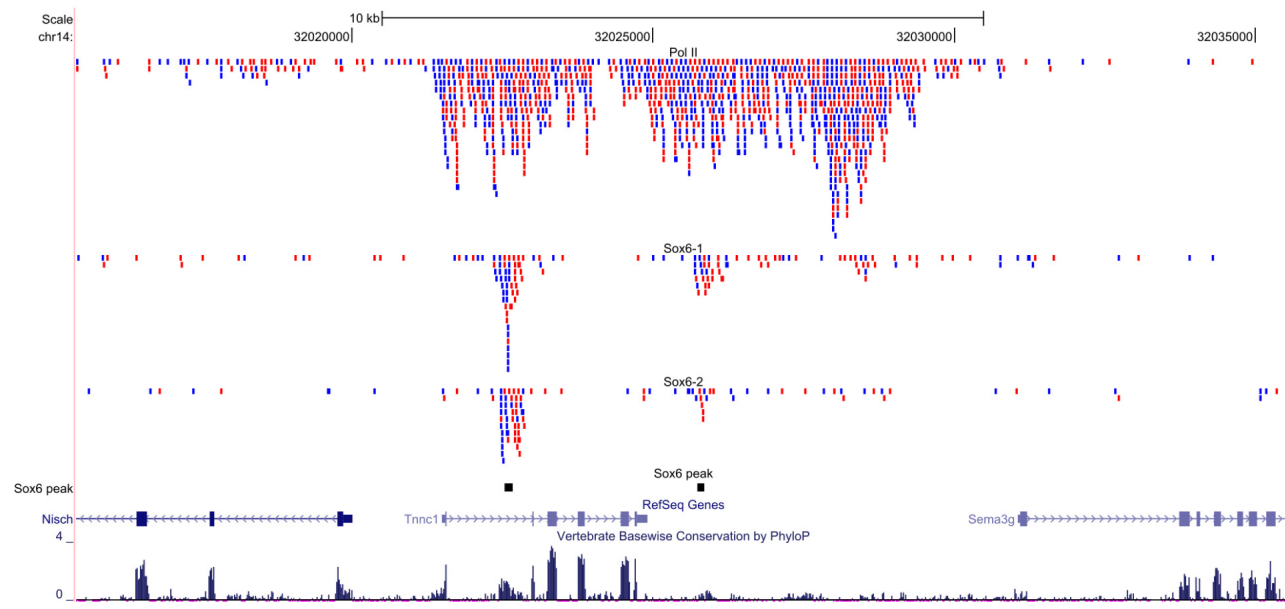

**E**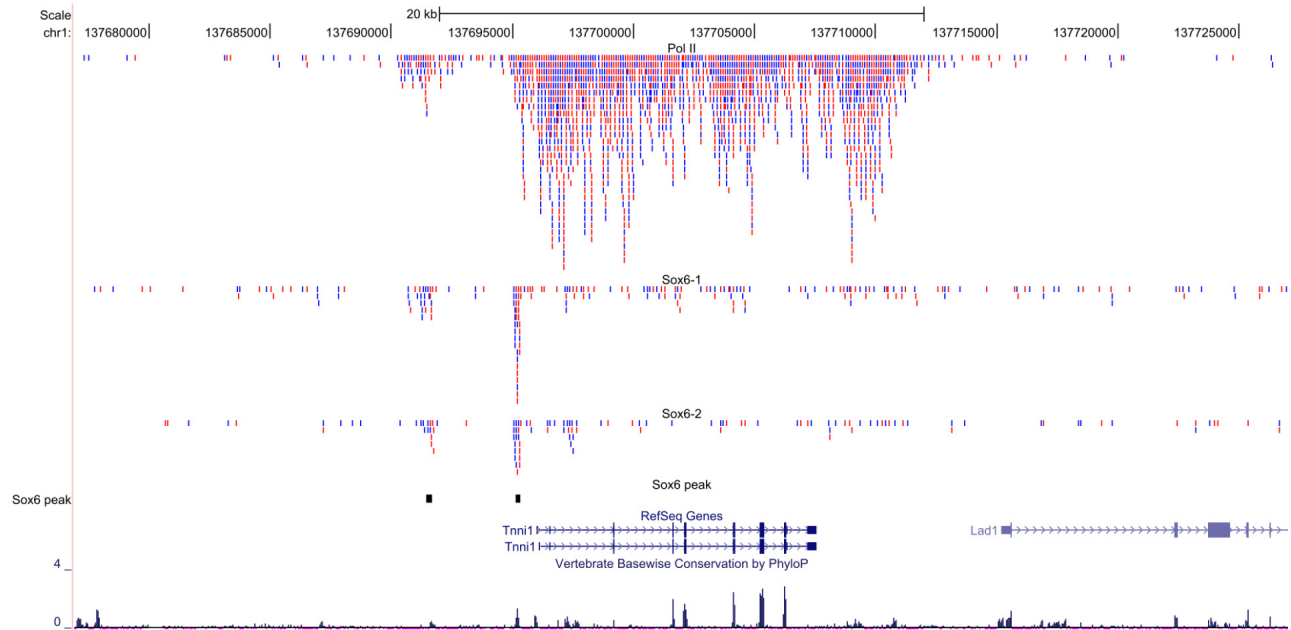**F**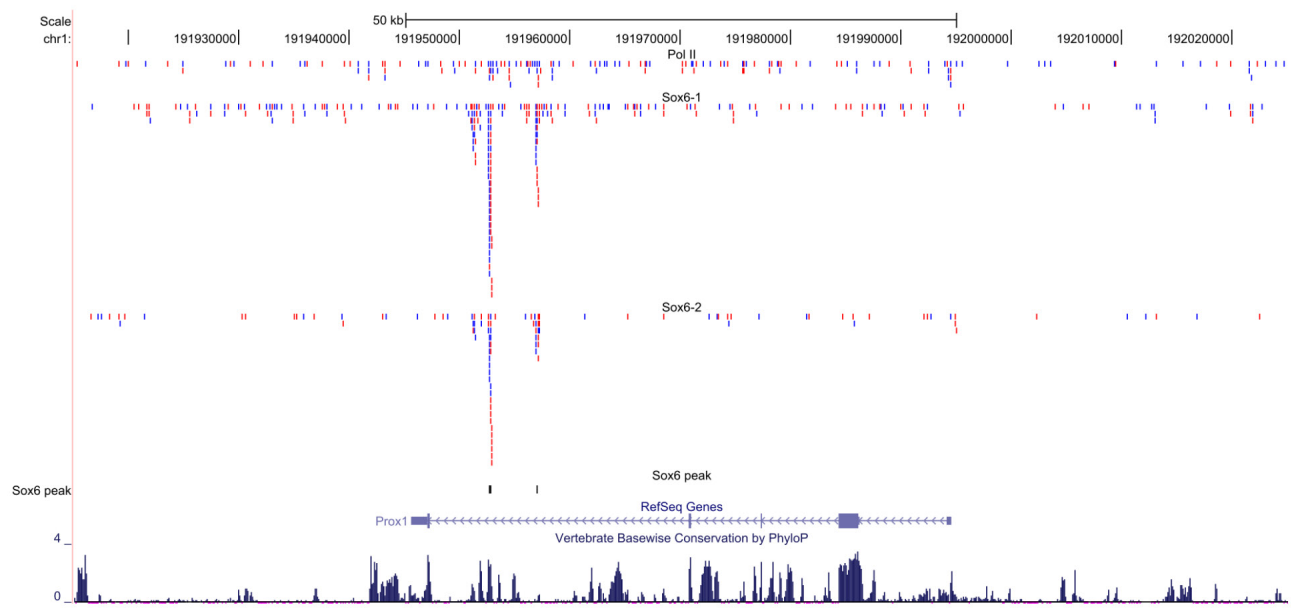

**G**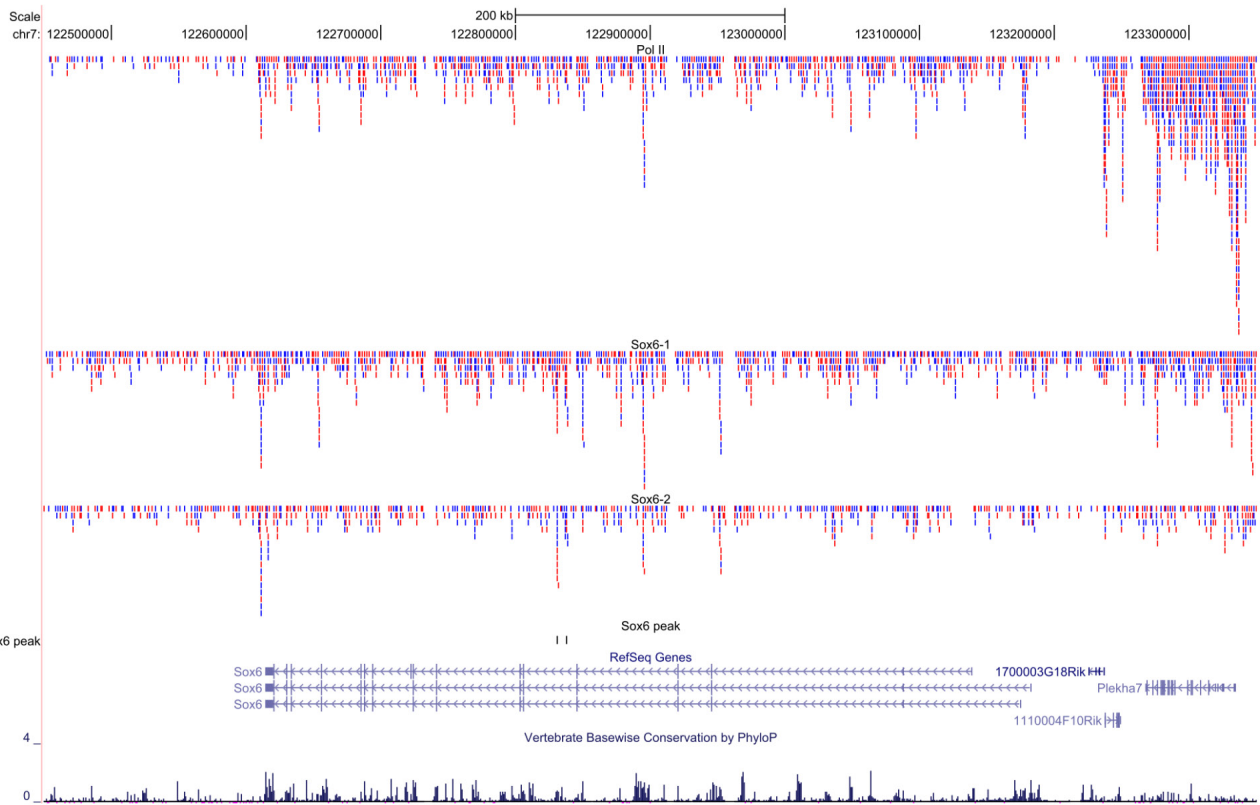

**H**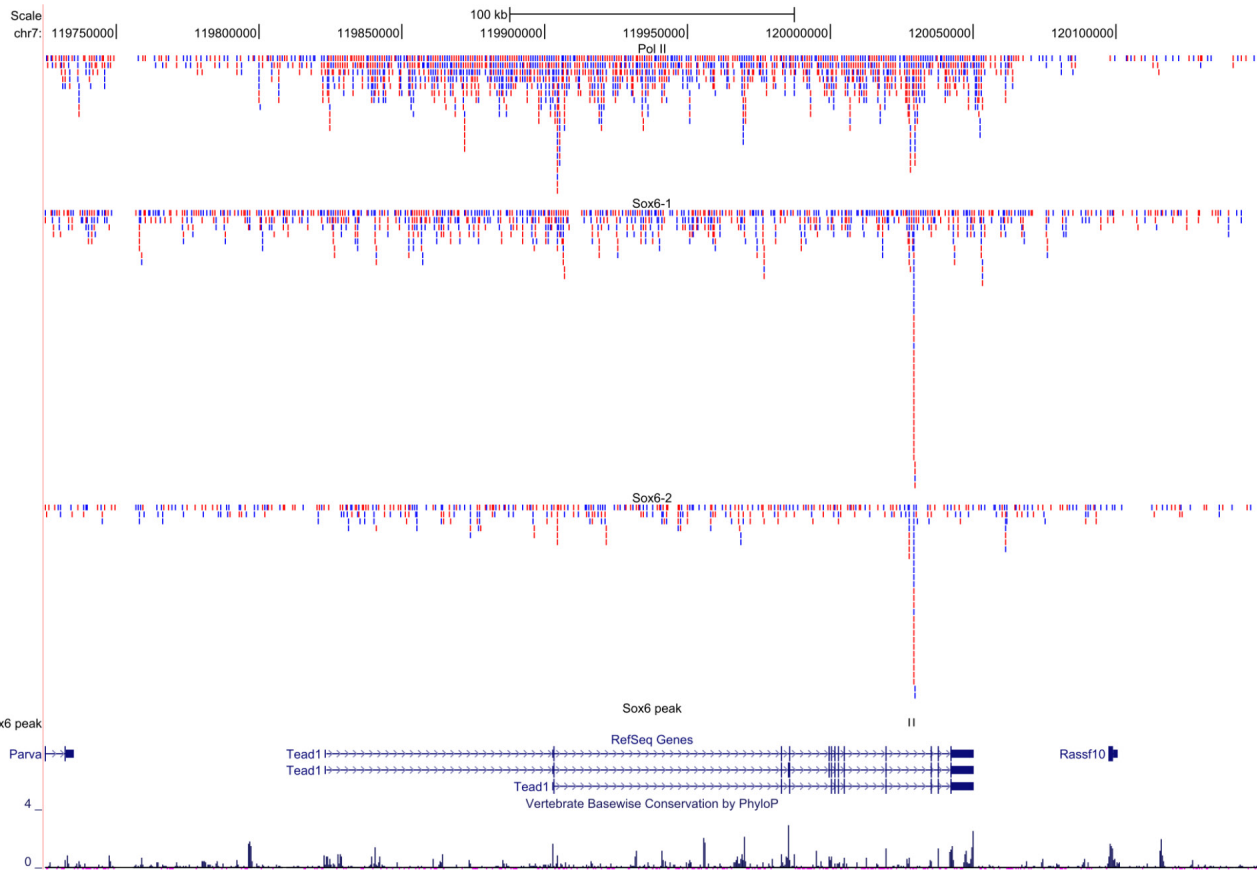

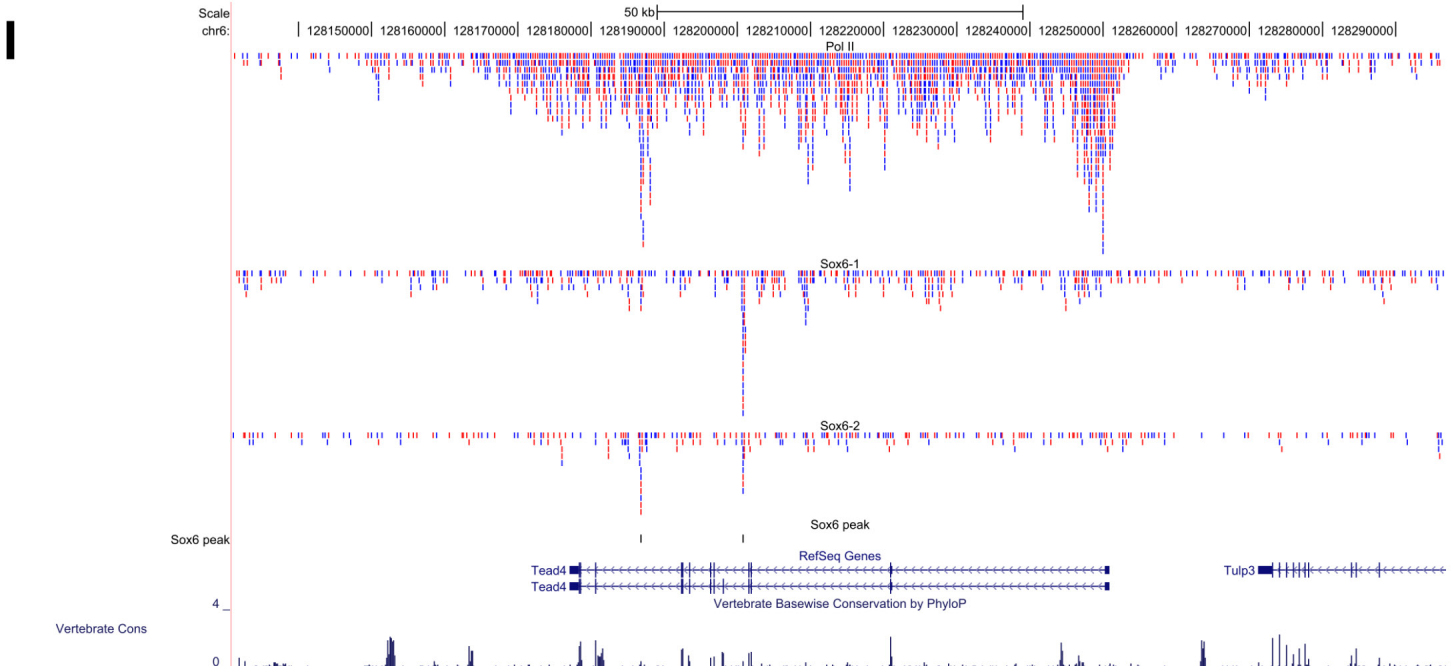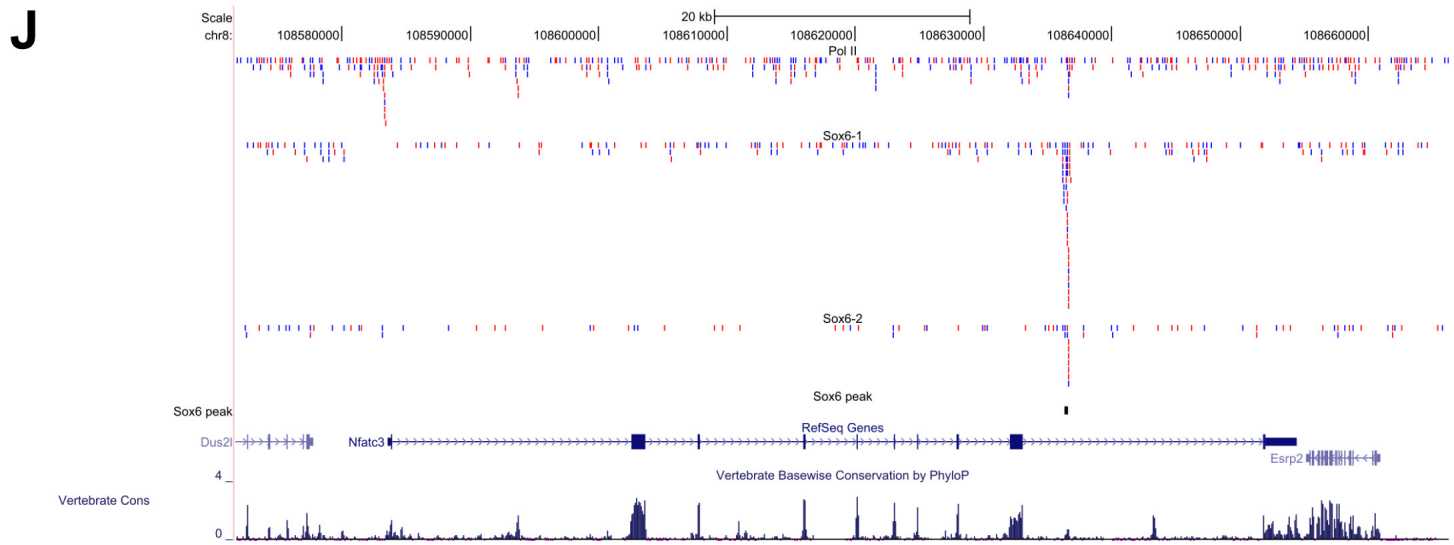

K

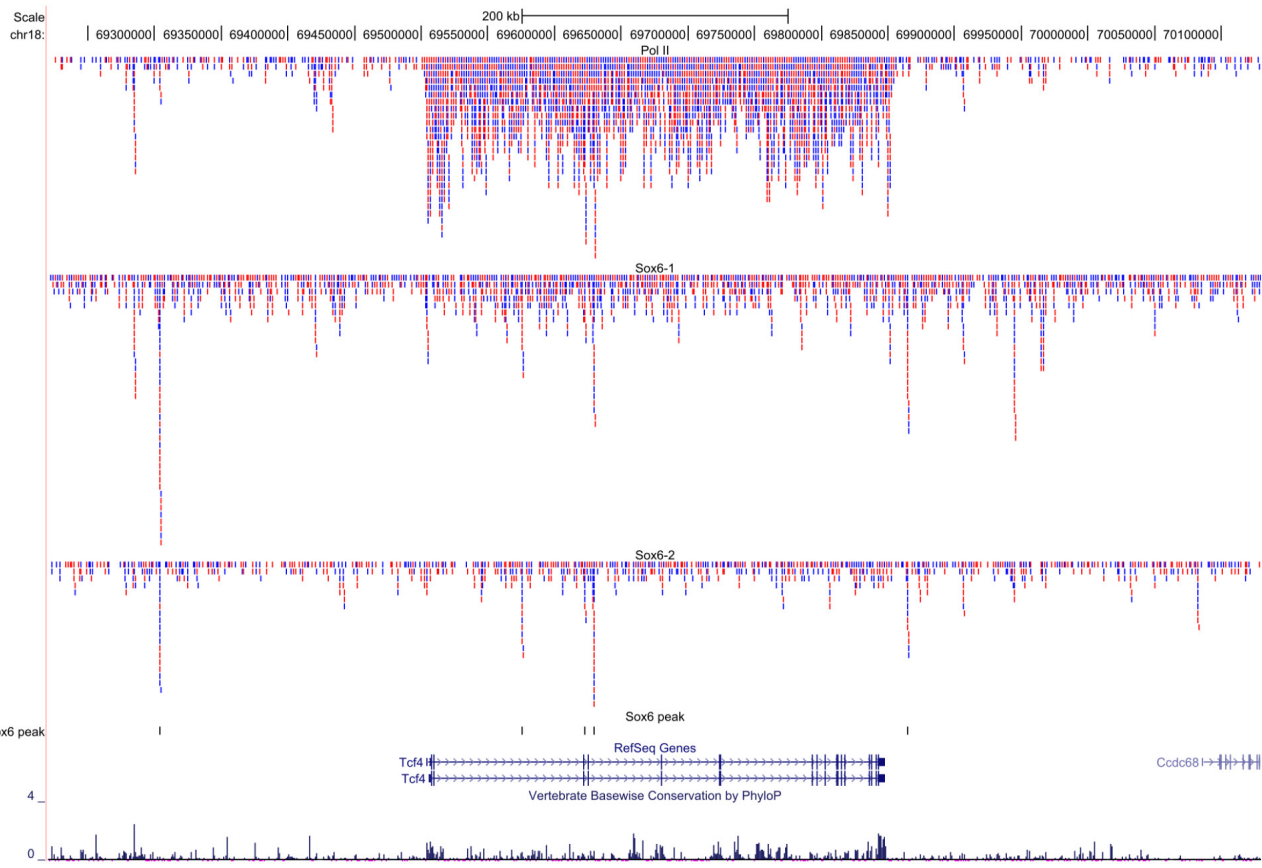

**L**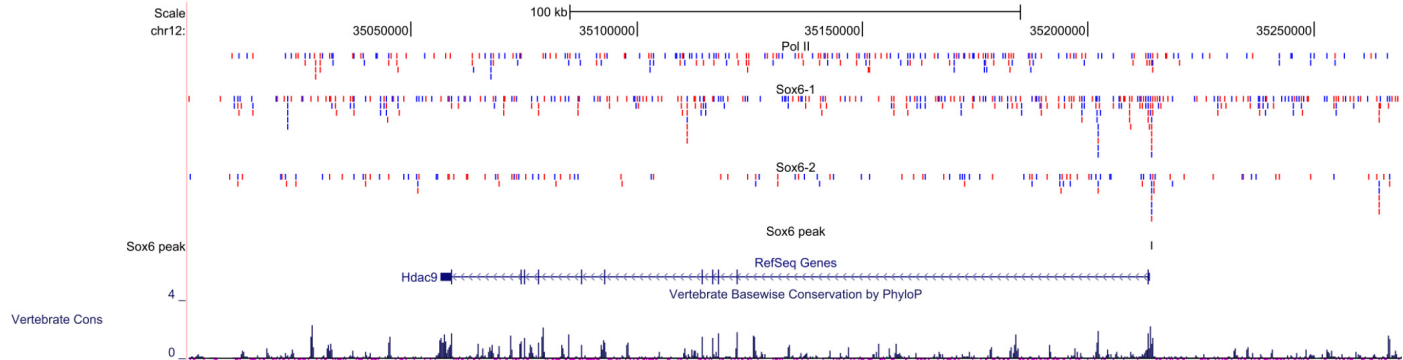**M**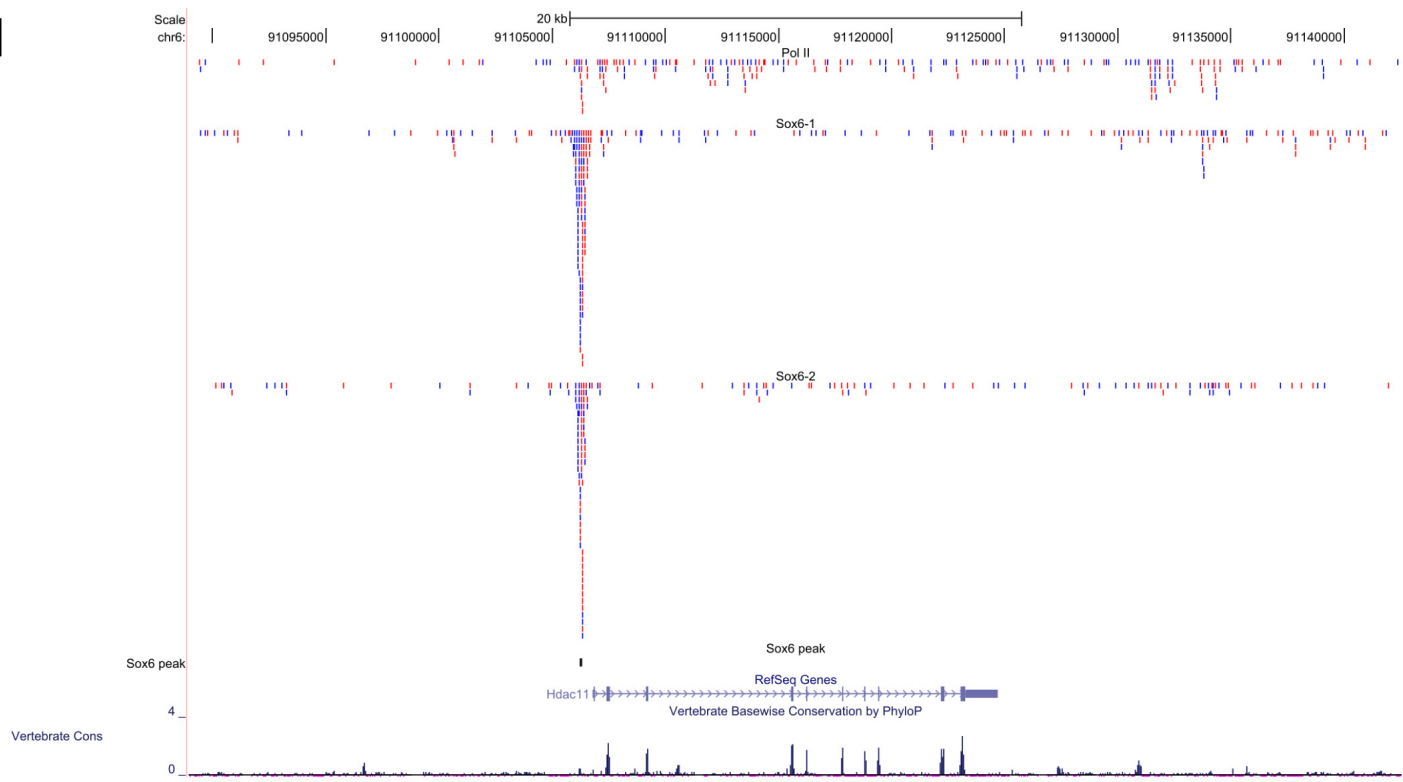

Supplement: Additional file 3 — Figure S2 Examples of Sox6 and Pol II binding events detected by ChIP-seq. ChIP-seq tracks from two data sets of Sox6 (Sox6-1 and Sox6-2) are shown together with Pol II track (Pol II) of the 2.8 million read data (see the Methods section for details). Common Sox6 binding peaks between the two data sets are indicated as black bars (Sox6 peak). Chromosomal positions (mouse NCBI37/mm9 assembly) as well as sequence conservation (Vertebrate Cons) are presented above and below the ChIP-seq plots, respectively. A. Myh1 and Myh2, B. Myh6 and Myh7, C. MyHC7b, D. Tnnc1, E. Tnni1, F. Prox1, G. Sox6, H. Tead1, I. Tead4, J. Nfatc3, K. Tcf4, L. Hdac9, and M. Hdac11. [file 1471-213X-11-59-S3.PDF]

**A**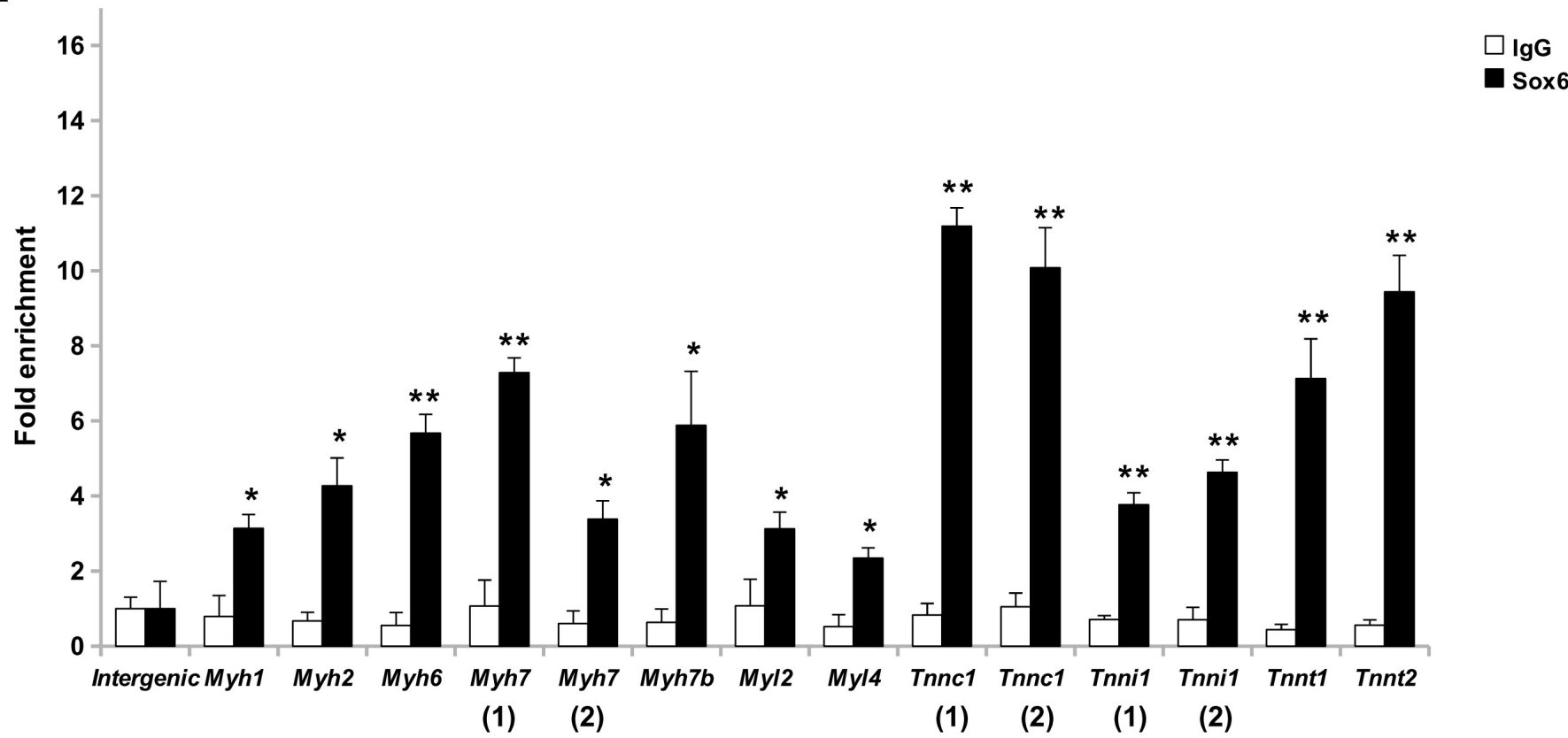

**B**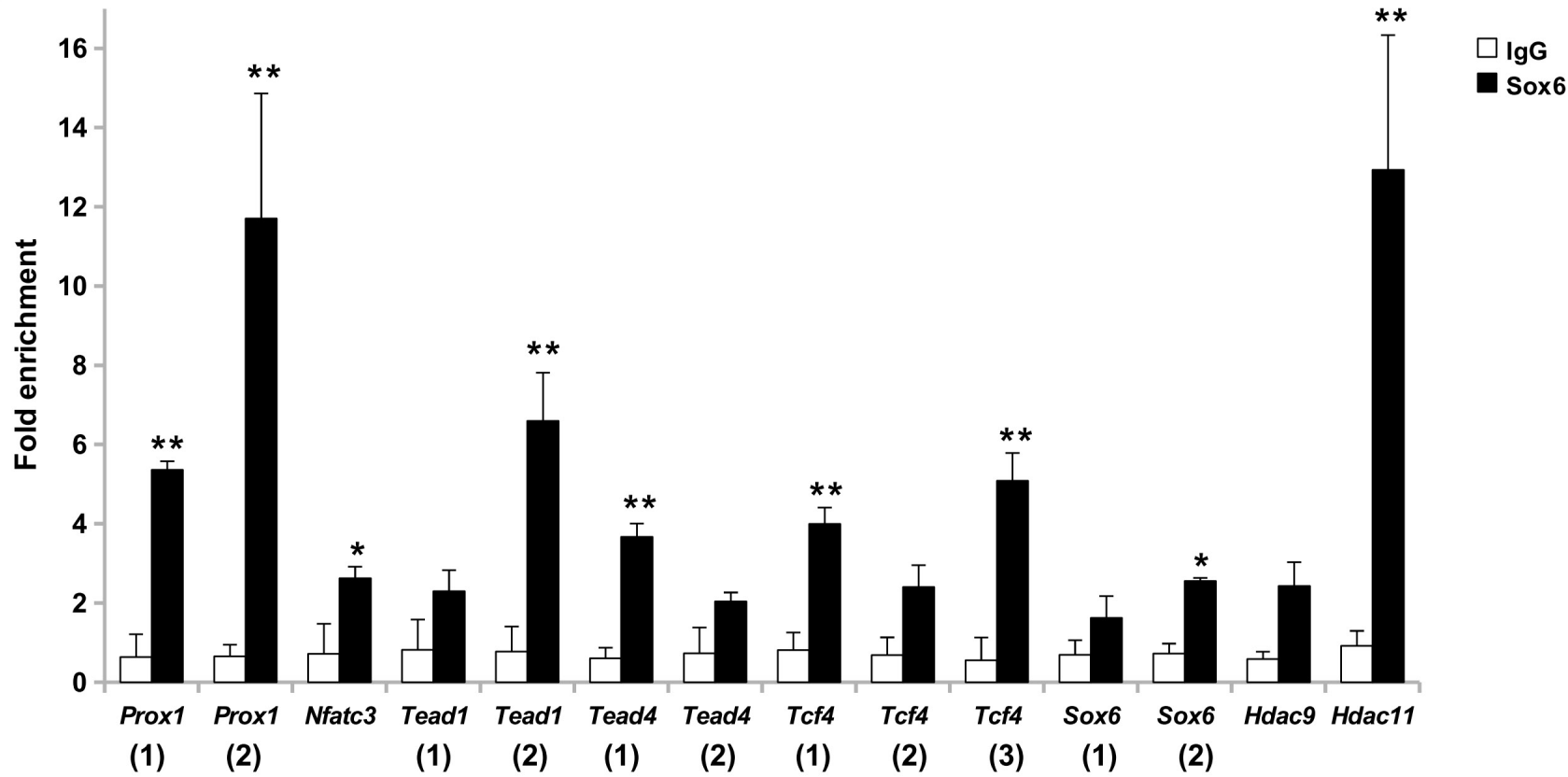

Supplement: Additional file 4 — Figure S3 Validation of Sox6 binding. A total of 28 Sox6 peaks identified for the 19 genes discussed in the text were verified by ChIP-qPCR (ChIP followed by quantitative PCR). The peak profiles are summarized in Additional file 3, Figure S2A-M. ChIP was performed using wild type myotubes and Sox6 antibody as described in the Methods section, and enrichment was quantified by qPCR using the primers designed to amplify each Sox6 binding site (Additional file 6, Table S3). As a negative control, an intergenic region without a Sox6 peak was used. Fold enrichment over a negative control region (Intergenic) are shown. The intergenic region showed no enrichment. Data are represented as mean ± SD (n = 3). (*) P < 0.05; (**) P < .005. A. Enrichment of the Sox6 binding sites associated with sarcomeric protein genes. B. Enrichment of the Sox binding sites associated with transcription regulatory genes. Numbers in the parentheses below gene symbol indicate relative positions (5' to 3') of multiple Sox6 binding sites associated to the gene. For Tcf4, only intragenic binding sites (see Additional file 3, Figure S2K) were tested. [file 1471-213X-11-59-S4.PDF]

**A**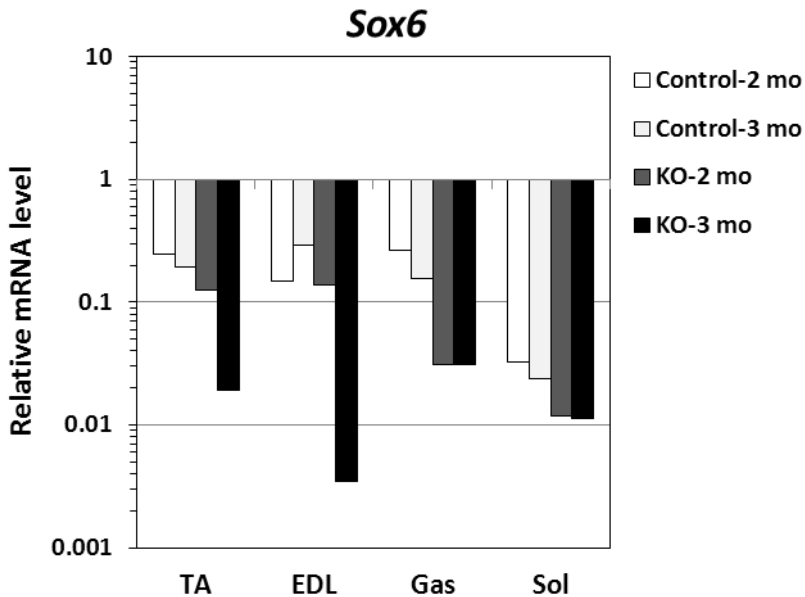

**B**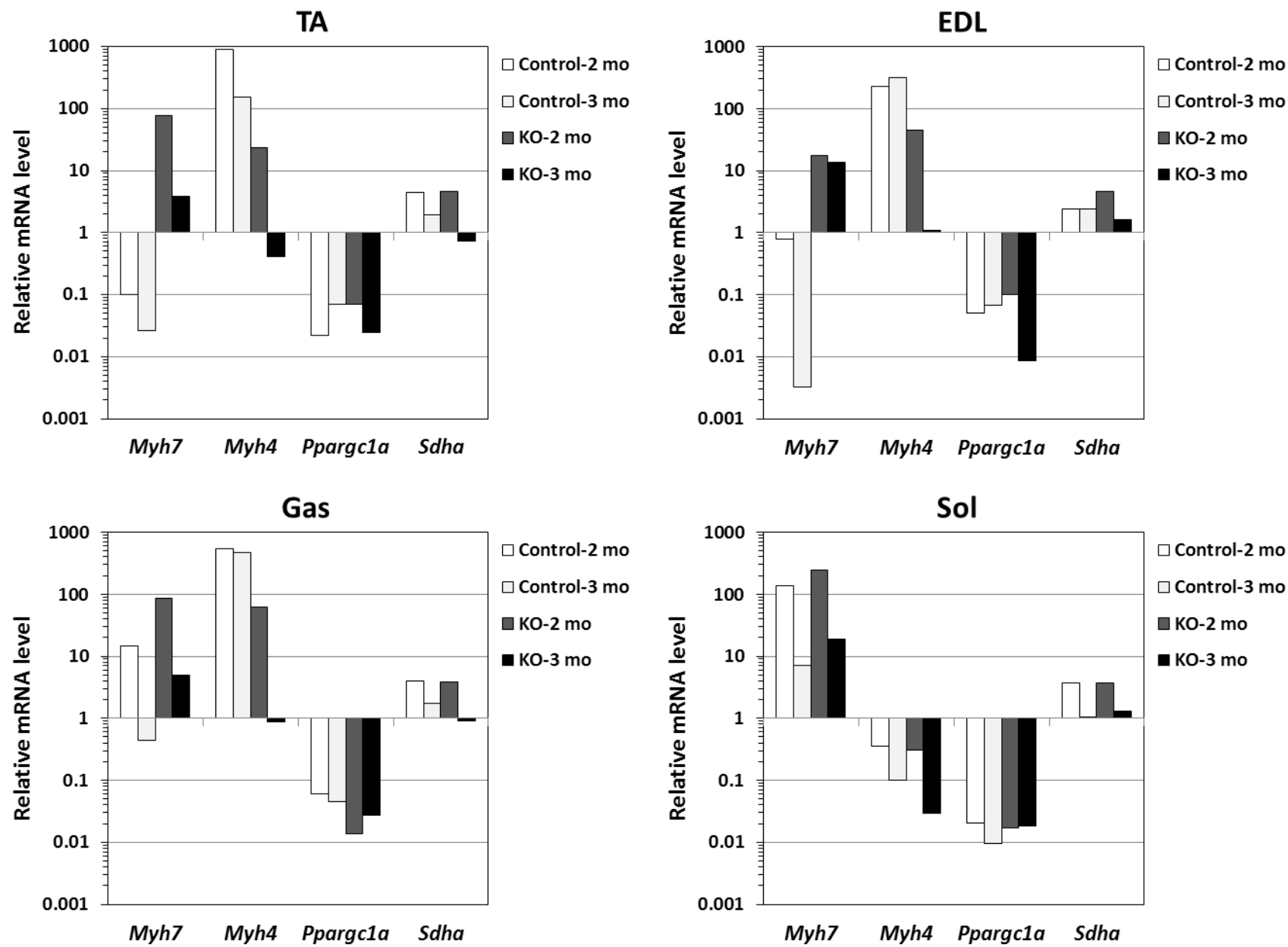

Supplement: Additional file 8 — Figure S4 Relative mRNA levels of the genes presented in Table 1. Relative mRNA levels against β-actin in TA, EDL, gastrocnemius (Gas), and soleus (Sol) of control (Sox6f/f) and Sox6 knockout (KO, Sox6f/f; Myf5-Cre) mice were calculated using the formula 2-ΔCt. A two and three month-old mice (2 mo and 3 mo, respectively) were analyzed. A. Relative mRNA level of Sox6. B. Relative mRNA level of Myh7, Myh4, Ppargc1a, and Sdha. [file 1471-213X-11-59-S8.PDF]

**A**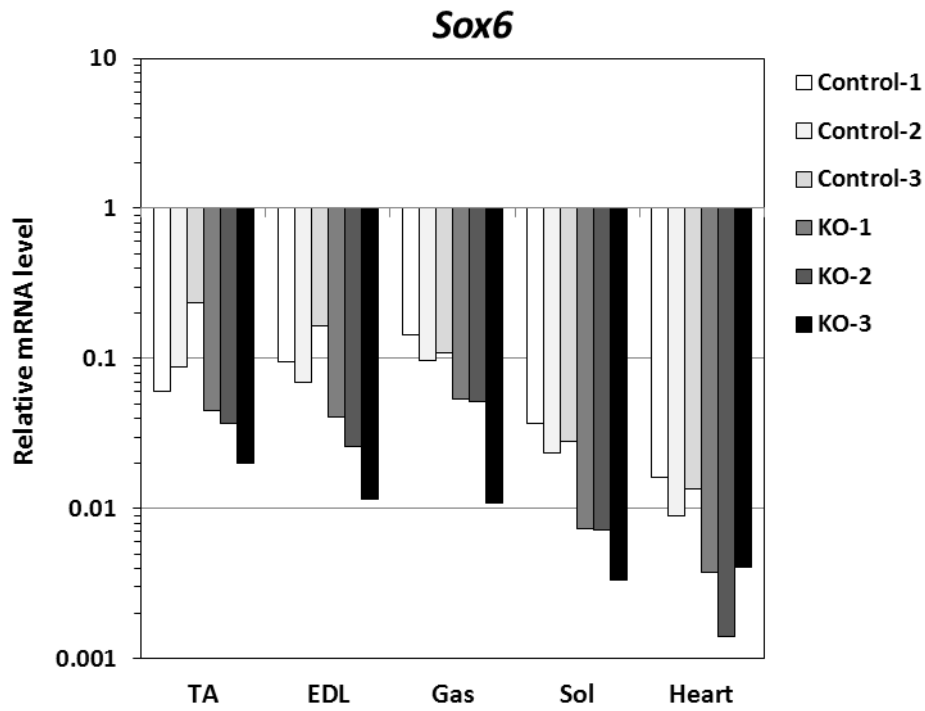

**B**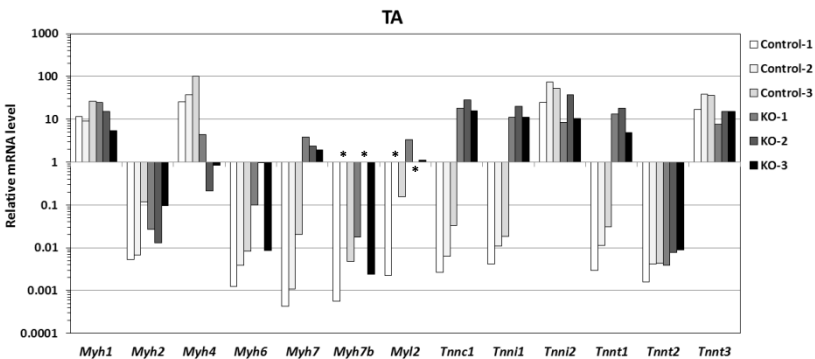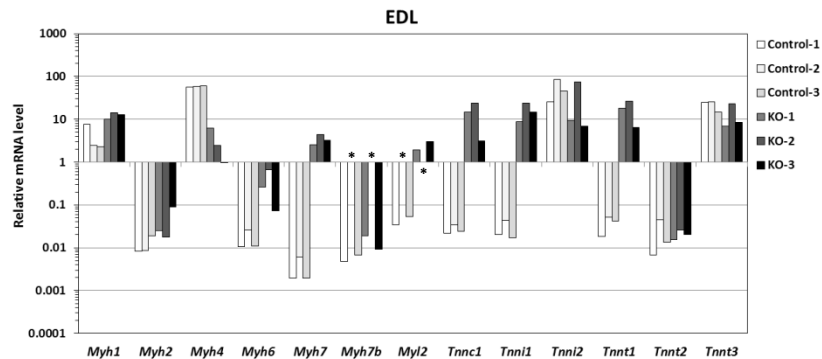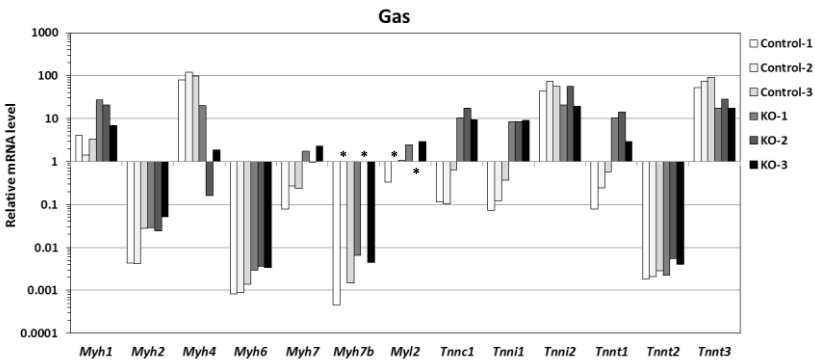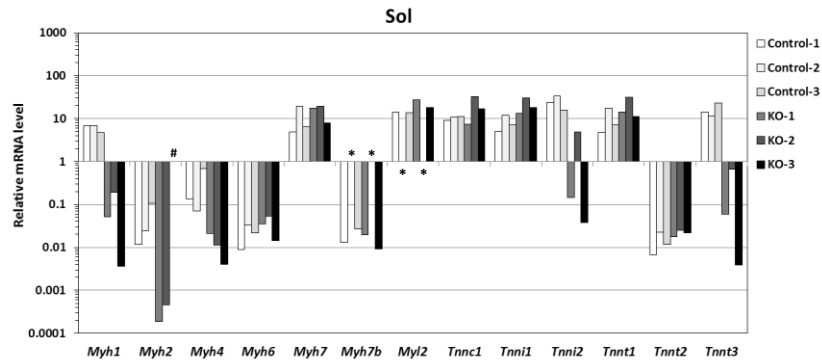

**C**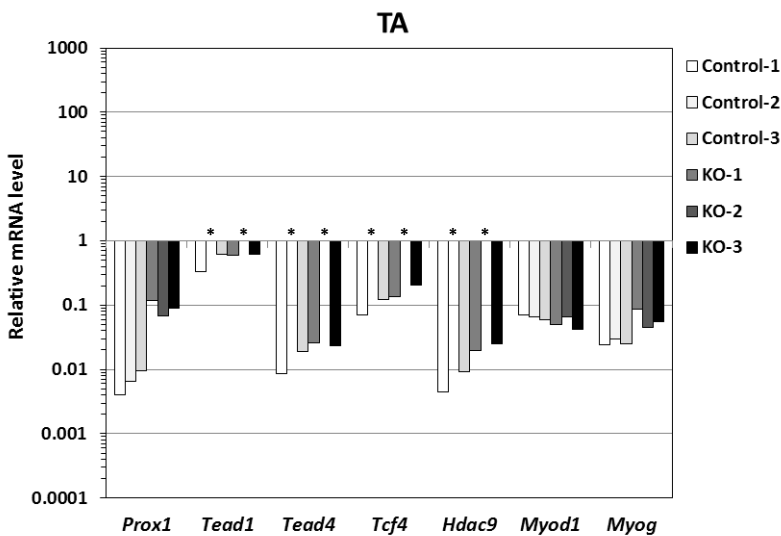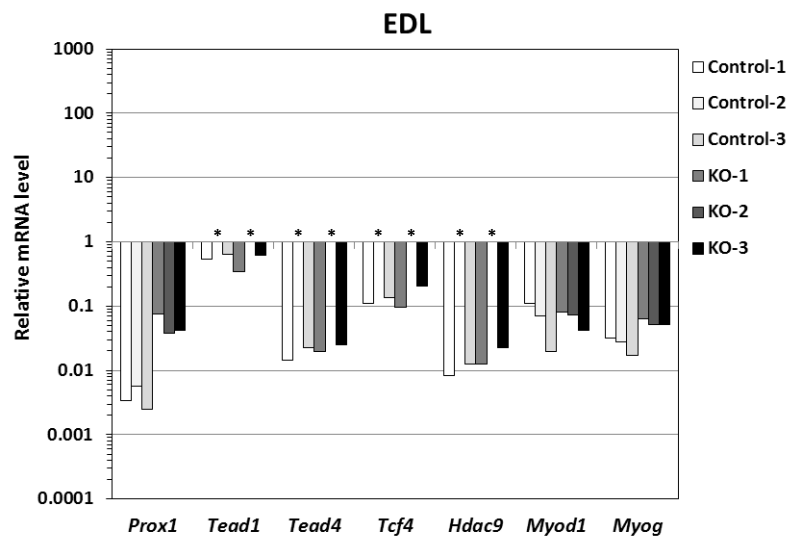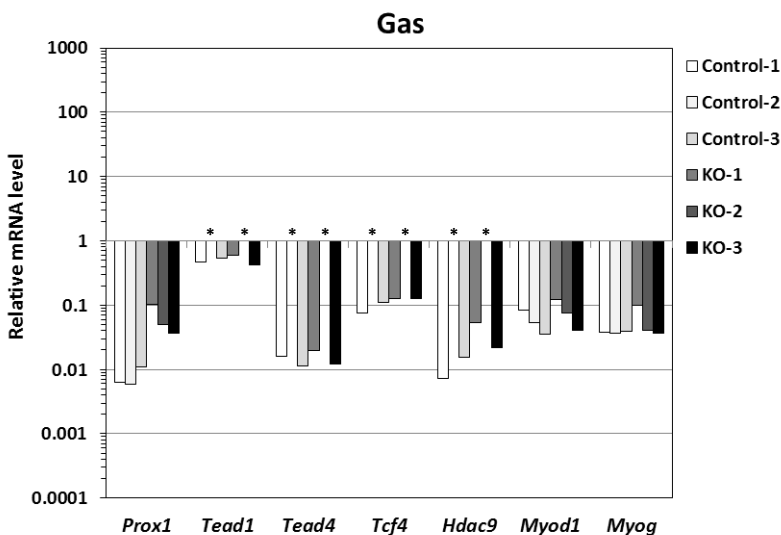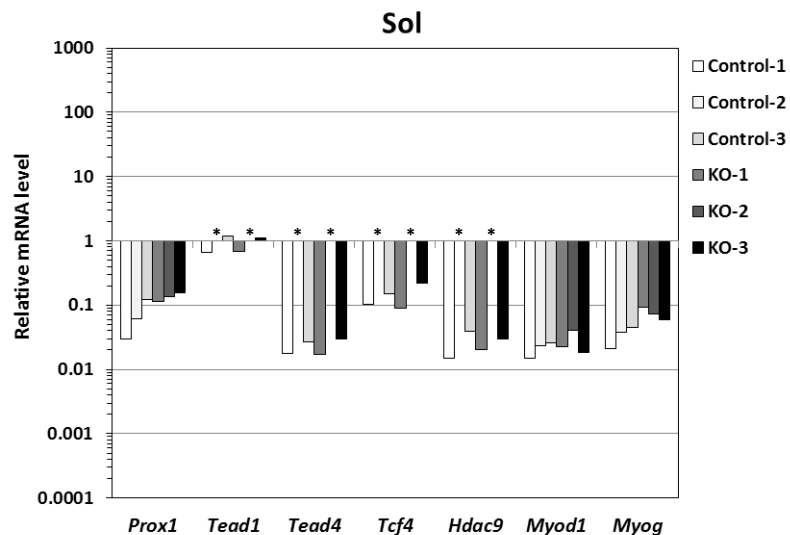

**D**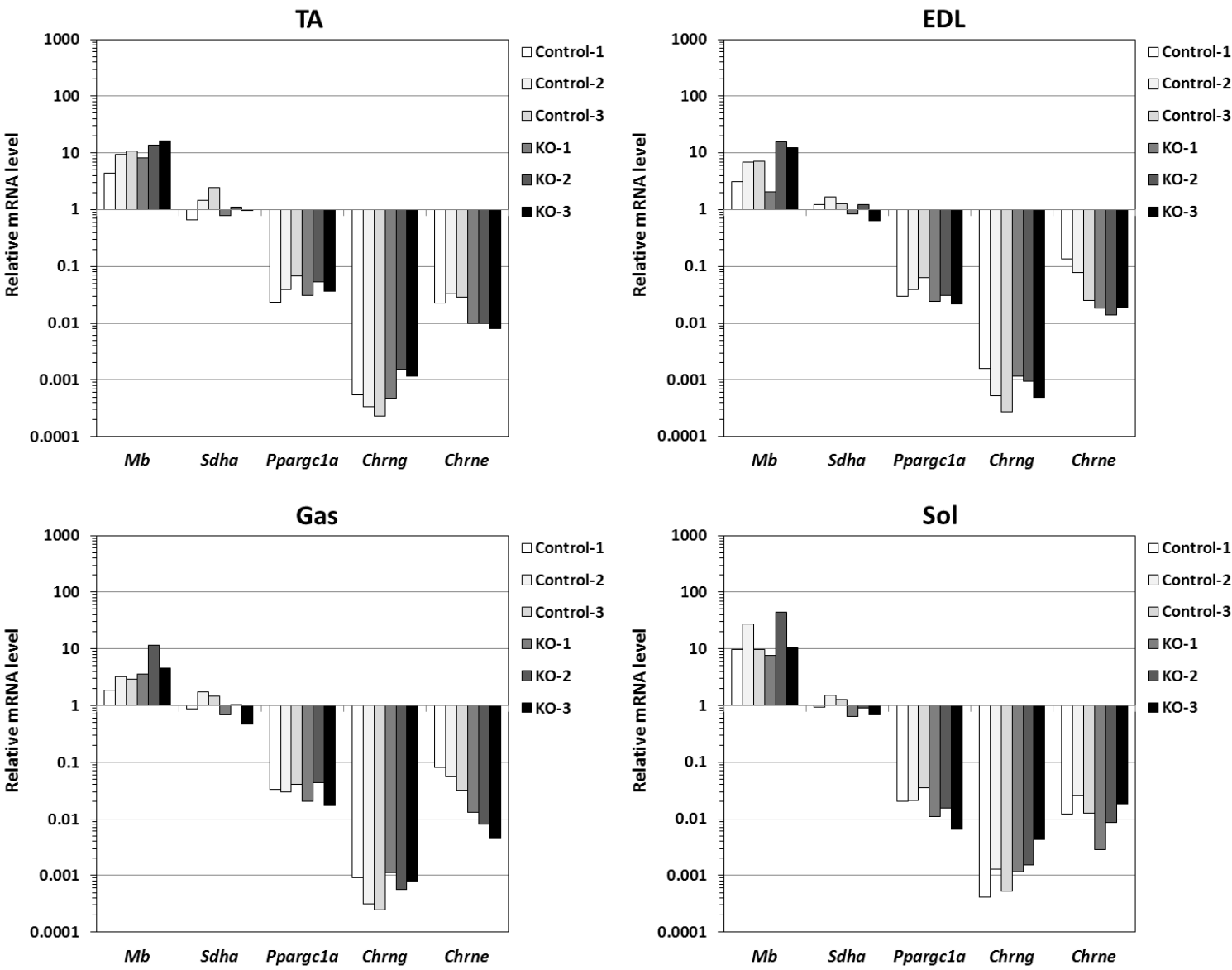

**F**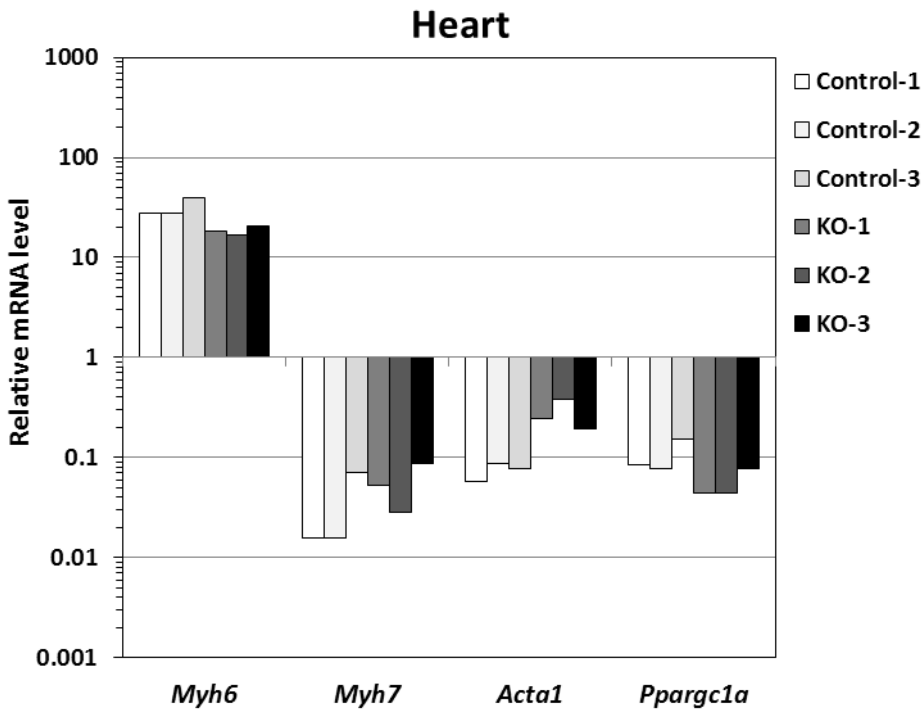

Supplement: Additional file 9 — Figure S5 Relative mRNA levels of the genes presented in Table 2and 4. Relative mRNA levels against β-actin in control (Sox6f/f) and Sox6 knockout (KO, Sox6f/f; MCK-Cre) mice were calculated using the formula 2-ΔCt. Two 2 month-old mice (mouse ID# 1 and 2) and one 3 old-month mouse (mouse ID# 3) were analyzed. A. Relative mRNA level of Sox6 in TA, EDL, gastrocnemius (Gas), soleus (Sol), and the heart. B. Relative mRNA level of contractile protein genes in TA, EDL, Gas, and Sol. C. Relative mRNA level of transcriptional regulatory genes in TA, EDL, Gas, and Sol. D. Relative mRNA level of metabolism related genes and acetylcholine receptor genes in TA, EDL, Gas, and Sol. E. Relative mRNA level of Myh6, Myh7, Acta1, and Ppargc1a in the heart. *: not determined. #: undetected. [file 1471-213X-11-59-S9.PDF]
